# Cancer-associated fibroblast-derived circKLHL24 drives perineural invasion in pancreatic cancer via dual regulation of the sec31a-CXCL12 axis

**DOI:** 10.1186/s13046-025-03489-2

**Published:** 2025-10-07

**Authors:** Tingting Li, Rihua He, Qing Tian, Tianhao Huang, Honghui Jiang, Huimou Chen, Yuan Yuan, Yong Jiang, Shangyou Zheng, Chonghui Hu, Shizong Li, Guolin Li, Rufu Chen

**Affiliations:** 1 School of medicine, South China University of Technology, Guangzhou, 510006 Guangdong China; 2 Department of Pancreas Center, Department of General Surgery, Guangdong Provincial People’s Hospital (Guangdong Academy of Medical Sciences), Southern Medical University, Guangzhou, 510080 Guangdong China; 3 Guangdong Provincial People’s Hospital (Guangdong Academy of Medical Sciences), Southern Medical University, Guangzhou, 510080 Guangdong China; 4 Guangdong cardiovascular Institute, Guangdong Provincial People’s Hospital, Guangdong Academy of Medical Sciences, Guangzhou, 510080 Guangdong China; 5 Department of Hepatobiliary and Pancreatic Surgery, Hainan General Hospital, Hainan Medical University Hainan Hospital, Haikou, 570311 Hainan China; 6 Department of Hepatobiliary Pancreatic and Splenic Surgery, The Sixth Affiliated Hospital, Sun Yat-sen University, Guangzhou, 510655 Guangdong China; 7 Department of Oncology, Sun Yat-sen Memorial Hospital of Sun Yat-sen University, Guangzhou, 510120 Guangdong China

**Keywords:** Cancer-associated fibroblasts, Circular RNAs, Perineural invasion, CXCL12

## Abstract

**Background:**

Cancer-associated fibroblasts (CAFs) are key drivers of neural invasion in pancreatic cancer, yet their regulatory mechanisms remain elusive.This study explores the role of circular RNAs (circRNAs) in CAFs and their involvement in regulating neural invasion in pancreatic cancer.

**Methods:**

CAF-derived circRNAs were identified through circRNA high-throughput sequencing and quantitative real-time PCR (qRT-PCR). The impact of CAF-derived circKLHL24 on perineural invasion (PNI) in tumor cells was evaluated both in vitro and in vivo. RNA sequencing, RNA pulldown, RNA immunoprecipitation, and luciferase reporter assays were conducted to identify downstream targets and elucidate the underlying mechanism of circKLHL24 in PNI.

**Results:**

CircKLHL24 (hsa_circ_0001369), a CAF-specific circRNA, is associated with PNI and poor survival in advanced PDAC. Silencing or overexpressing circKLHL24 in CAFs altered the ability of CAFs to induce tumor cell invasion and nerve infiltration via chemokine (C-X-C Motif) ligand 12 (CXCL12). Mechanistically, first, circKLHL24 binds to the membrane protein Sec31A, inhibiting its ubiquitination and degradation, thereby enhancing CXCL12 secretion. Second, circKLHL24 acts as a sponge for miR-615-5p, relieving its suppression of CXCL12 mRNA and amplifying CXCL12 expression. Moreover, high circKLHL24 levels were positively correlated with elevated serum CXCL12 levels in PDAC and poor patient survival. Targeting circKLHL24 or neutralizing CXCL12 suppresses PDAC invasion and neuronal recruitment in nude mouse and KPC models.

**Conclusions:**

The circKLHL24/Sec31A/miR-615-5p/CXCL12 axis is critical for CAF-induced PNI in PDAC. Therefore, circKLHL24 could serve as a potential therapeutic target for PDAC.

**Supplementary Information:**

The online version contains supplementary material available at 10.1186/s13046-025-03489-2.

## Background

Pancreatic ductal adenocarcinoma (PDAC) is a highly aggressive malignancy characterized by a propensity for early metastasis and poor prognosis [1]. A hallmark of PDAC is perineural invasion (PNI), in which cancer cells infiltrate surrounding nerves, contributing to severe pain and increased recurrence rates [2]. PNI is observed in approximately 80–100% of PDAC cases and is associated with diminished overall survival [3]. In our previous randomized trial, the incorporation of modified extended retroperitoneal nerve dissection into standard pancreaticoduodenectomy resulted in a 73.3%% increase in median disease-free survival (23.4 vs. 13.5 months, *p* < 0.001) among patients with pancreatic head carcinoma and preoperative CA19-9 levels below 200.0 U/mL [4]. Notably, this surgical approach demonstrated superior efficacy in mitigating neurogenic pain syndromes post-resection, providing clinical validation for PNI-directed therapeutic strategies. Emerging evidence suggests that tumor microenvironment (TME) reprogramming and PNI development exhibit spatiotemporal coupling during progression, with molecular crosstalk between stromal activation and neuroplasticity pathways potentially driving this co-evolutionary process [5, 6]. These observations position stromal-nerve axis modulation as a critical frontier in PDAC biology.

The pancreatic TME is a dynamic ecosystem dominated by cancer-associated fibroblasts (CAFs), that orchestrate tumor progression through extracellular matrix remodeling and cytokine secretion [7]. Studies show that stroma-derived leukemia inhibitory factor (LIF) in the pancreatic TME fosters Schwann cell-mediated neural plasticity through paracrine signaling [8]. Building on these findings, our group discovered that CAFs release the long non-coding RNA PIAT via extracellular vesicles, creating autocrine–epigenetic loops that sustain YBX1-mediated RNA methylation and pro-invasion transcriptome shifts in recipient tumor cells [9]. Stromal cell-derived CXCL12, a chemokine critical for cell migration and survival, has been implicated in limiting the efficacy of immunotherapy in PDAC [10, 11]. In advanced pancreatic cancer, CXCL12 blockade with NOX-A12 plus checkpoint inhibition was safe, resulted in prolonged survival, and boosted T-cell infiltration, indicating a promising immunomodulatory approach [12]. Despite these advances, the contribution of CAF-secreted CXCL12 to PNI in PDAC remains uncharacterized.

Circular RNAs (circRNAs) represent a unique RNA subclass characterized by covalently closed-loop structures formed through a back-splicing mechanism, conferring topological stability and resistance to RNA exonucleases [13, 14]. Our research team has systematically deciphered CAF-derived circRNA networks in pancreatic cancer progression. We demonstrated that circCUL2 drives inflammatory CAF differentiation via the MyD88/NF-κB/IL6 axis, establishing autocrine-paracrine circuits that accelerate tumor-stroma crosstalk [15]. Moreover, we demonstrated that CAF-derived circFARP1confers gemcitabine resistance by stabilizing CAV1 and activating the LIF/STAT3 signaling pathway [16], while circBIRC6, packaged in extracellular vesicles (EVs), drives platinum resistance via XRCC4 SUMOylation–mediated DNA repair [17], underscoring the critical role of circRNAs in pancreatic cancer chemoresistance. While these studies establish CAFs-derived circRNAs as architects of stromal inflammation and chemoresistance, their role in orchestrating neurotropic behaviors—particularly through secretory chemokine networks like CXCL12—remains unexplored.

This study unveils a novel mechanism by which CAF-derived circKLHL24 drives PNI in PDAC through dual regulation of the Sec31A-CXCL12 axis. First, circKLHL24 binds to the membrane protein Sec31A, preventing its ubiquitination and degradation, thereby promoting CXCL12 secretion. Second, circKLHL24 acts as a sponge for miR-615-5p, relieving its suppression of CXCL12 mRNA and amplifying CXCL12 expression. This synergistic mechanism elevates CXCL12 levels in the TME, promoting cancer cell invasion, neuronal recruitment, and PNI. Our work bridges the gap between circRNA biology, CAF function, and neural invasion, and proposes a new paradigm for targeting stroma-mediated signaling in PDAC.

## Methods

### Patients and clinical samples

Tumor samples were collected from 74 patients diagnosed with pancreatic ductal adenocarcinoma (PDAC) who underwent surgical resection at Guangdong Provincial People’s Hospital between 2017 and 2024. None of the patients had received prior radiotherapy, chemotherapy, immunotherapy, or targeted therapy before surgery. Patients lost to follow-up were excluded from the study. The cohort consisted of both male and female patients, with no gender-based restrictions for the inclusion of clinical samples. Overall survival (OS) was defined as the time from randomization to death or the last follow-up (December 2024), while disease-free survival (DFS) was the time interval from randomization to the first occurrence of disease recurrence or failure. Perineural invasion (PNI) was identified when cancer cells encased at least 33% of the nerve circumference or infiltrated the nerve sheath’s epineural, perineurial, or endoneurial compartments. PNI severity was assessed based on two parameters: the extent of nerve involvement and its frequency. The extent of PNI was graded as 0 (no involvement), 1 (perineural, cancer cells encasing at least 33% of the nerve), or 2 (intraneural, cancer cells infiltrating the nerve sheath). The frequency of PNI was classified as 0 (absent), 1 (low), 2 (moderate), or 3 (high). A composite severity score, calculated by multiplying the extent and frequency scores, ranged from 0 to 6. Patients with a score of 4–6 were classified as PNI-positive (PNI+), while those with scores of 0–3 were categorized as PNI-negative (PNI−). All patients provided informed consent, and the study procedures were approved by the Ethical Committee of the respective hospitals.

### Primary cell isolation and culture

Cancer-associated fibroblasts (CAFs) and primary normal fibroblasts (NFs) were isolated from pancreatic ductal carcinoma (PDAC) and adjacent non-cancerous tissues. Fresh surgical resections were collected with informed consent from Guangdong Provincial People’s Hospital. For CAF isolation, tumor samples (approximately 0.5 cm²) were carefully excised from the core of PDAC tissues using a sterile scalpel. A portion of the isolated tissue was reserved for histological analysis to confirm the diagnosis, while the remaining tissue was minced and digested in a collagenase digestion medium (DMEM/F12, 125 units/mg collagenase, 10 mg/mL insulin, 0.5 mg/mL hydrocortisone, 100 U/mL penicillin, and 100 mg/mL streptomycin) for 30 min. The digestion was stopped by adding 10% FBS/DMEM, and the dissociated tissue was incubated for 10 min at 37 °C without shaking on a 6-cm dish. The stromal cell-enriched supernatant was then collected, centrifuged at 250 g for 5 min, and the fibroblasts were cultured. For NF isolation, similar procedures were followed, with normal adjacent pancreatic tissue collected from areas at least 3 cm away from the primary PDAC tumors. All samples were obtained from surgical resections, not intraoperative biopsies. Primary fibroblasts were cultured in Dulbecco’s Modified Eagle’s Medium (DMEM, Gibco) with 15% fetal bovine serum (FBS, Gibco) and 1% penicillin/streptomycin at 37 °C in a humidified atmosphere containing 5% CO_2_. For culturing human PDAC cell lines, MiaPaCa-2 cells were purchased from the American Type Culture Collection (ATCC, Manassas, VA), and were maintained in DMEM supplemented with 10% fetal bovine serum at 37 °C with 5% CO_2_.

### Western blotting

Protein was extracted from the cells using RIPA lysis buffer (CWBIO, China), and then subjected to SDS-PAGE. The proteins were transferred to polyvinylidene difluoride (PVDF) membranes. The membranes were incubated with primary antibodies against CXCL12, Sec31A, GAPDH, p-ERK1/2, ERK1/2, β3-Tubulin, pMEK1/2, MEK1/2, Snail, N-cadherin, p-AKT, AKT, and p-PI3K. HRP-conjugated secondary antibodies were applied, and the immunoreactive bands were detected using the ECL detection system (Millipore, Germany) and photographed with a Chemi XT4 imaging system. A list of all antibodies used can be found in Supplementary Table S1.

### RNA isolation and quantitative real-time PCR (qRT–PCR)

Total RNA was extracted using TRIzol reagent (Life, USA) and reverse-transcribed with the PrimeScript RT Reagent Kit (DRR037A, Takara, Japan). The resulting cDNA was then amplified by quantitative reverse transcription PCR (qRT–PCR) on a Light Cycler 480 Detection System (Roche, Switzerland) using the TB Green Premix Ex Taq™ kit (RR820A, Takara, Japan), with GAPDH or U6 serving as internal controls. The relative gene expression levels in cells were calculated using the 2−∆∆CT method. For tissue expression analysis, gene expression levels were normalized to GAPDH expression using the ∆CT method. To assess the clinical significance of circKLHL24 and miR-615-5p, tissue samples from 74 PDAC patients were divided into two groups—low and high expression. Samples with normalized expression levels (∆CT) below or equal to the median value were classified as the low expression group, while those with levels above the median were classified as the high expression group. This was done by performing a tenfold gradient dilution of the reference standard. Using the measured cycle threshold (CT) values of the standards and their known concentrations, a standard curve for log (copy number) versus CT values was constructed. The standard curve was then used to extrapolate the number of circKLHL24 molecules in NFs and CAFs. All primers used are listed in Supplementary Table S2.

### RNase R digestion and actinomycin D assay

For the RNase R digestion assay, total RNA from CAFs was treated with or without 5 U/µg RNase R (RNR07250, Epicenter Technologies) and incubated at 37 °C for 30 min. In the actinomycin D assay, total RNA was treated with 2 µg/mL actinomycin D (Sigma, USA) for various time points (0, 4, 8, 12, and 24 h). The expression levels of circKLHL24 and KLHL24 were measured by qRT–PCR. All experiments were performed in triplicate.

### Plasmid construction and transfection

CircKLHL24 was inserted into the pCD-ciR vector by IGE (Guangzhou, China). Luciferase reporter plasmids for circKLHL24, its untranslated region (UTR), and mutant versions of the luciferase reporters were synthesized by IGE (Guangzhou, China). shRNA and miRNA mimics or inhibitors were obtained from IGE (Guangzhou, China). Transfections of plasmids and oligonucleotides were carried out using Lipofectamine 3000 (Invitrogen, USA), in accordance with the manufacturer’s protocol. The targeted sequences of the oligonucleotides are provided in Supplementary Table S3.

### Conditioned medium preparation

Primary fibroblasts (2 × 10⁶) were plated in a 10 cm² culture dish and cultured in FM medium. Once the cells reached 70% confluence, they were washed twice with PBS and then cultured in fresh DMEM containing 10% fetal bovine serum (SBI, USA) for 48 h. The conditioned medium (10⁵ cells/ml) was then collected and centrifuged at 3000 × g for 10 min to remove cells and debris. The supernatant was either used immediately or stored at -80 °C for subsequent use in stimulating cancer cells (at a concentration of 5 × 10⁵ cells/ml) or DRG cells.

### Transwell assays

The pretreated cells were seeded in the top chamber with 200 µl of serum-free medium, with or without Matrigel (Matrigel, BD Biosciences, NY, USA). Complete medium (700 µl) was added to the bottom compartment. After 12 h of incubation, the cells on the upper surface of the top chamber were removed, and the invading cells were fixed and stained with crystal violet. The number of invaded cells was counted and images were captured using a light microscope. Three independent experiments were conducted. In some cases, Miapca2 was treated with neutralizing anti-CXCL12 monoclonal antibodies (120 µg/ml, cat. MAB310, R&D ).

### Neural invasion in vitro model

For 3D co-culture of tumor cells and DRG analysis, DRG isolated from newborn mice was seeded on a 6-well plate with 20 µL Matrigel (Invitrogen, USA). PANC-1 cells were seeded beside approximately 2 mm the DRG in 20 µl Matrigel. After solidification, CAF-conditional medium or DMEM was added and renewed every 2 days. The phase-contrast image was taken at day 1 and day 7 by a light microscope (Invitrogen, USA). The neural invasion index = 1- (The nearest distance of tumor cell to DRG at day 7) / (The nearest distance of tumor cell to DRG at day 7). In some cases, Miapca2 was treated with neutralizing anti-CXCL12 monoclonal antibodies (120 µg/ml, cat. MAB310, R&D).

### Isolation of cytoplasmic and nuclear RNA

Cytoplasmic and nuclear RNA from CAFs was isolated using the NE-PER Nuclear and Cytoplasmic Extraction Reagents (Thermo Scientific, USA) following the manufacturer’s instructions. The ratio of cytoplasmic to nuclear RNA was then determined by qRT–PCR. U6 was used as the nuclear control, while GAPDH served as the cytoplasmic control.

### Multiplex immunofluorescence (mIF)

Multiplex immunofluorescence staining was performed using the PANO 4-plex IHC Kit (Panovue, China) following the manufacturer’s instructions. Briefly, after deparaffinization and rehydration, paraffin slides were subjected to heat-induced antigen retrieval in EDTA buffer (pH 9.0) using a microwave. The slides were then incubated with goat blocking serum (Golden Bridge Biological Technology, China) for 10 min at room temperature, followed by incubation with primary antibodies against CXCL12 and FAP. Afterward, horseradish peroxidase-conjugated secondary antibodies were applied, and tyramide signal amplification was performed. The slides were microwaved after each tyramide amplification step. DAPI was used to stain the nuclei. Images were captured using a Nikon 80i microscope.

### Fluorescence in situ hybridization (FISH)

FISH was carried out using an In Situ Hybridization Kit (Gene Pharma, Guangzhou, China) following the manufacturer’s instructions. Cy3-labeled circKLHL24, FAM-labeled hsa-miR-615-5p, and FAM-labeled Sec31A probes were hybridized with cells overnight at 37 °C. All images were captured using confocal microscopy. The targeted sequences of the probes are listed in Supplementary Table S2.

### RNA in situ hybridization (ISH)

The expression of circKLHL24 in PDAC tissues was assessed by ISH analysis using an ISH Detection Kit (MK1032, BOSTER, China) according to the manufacturer’s instructions. Briefly, after deparaffinization, rehydration, and digestion, specimens were incubated with digoxin-labeled circKLHL24 probes for 18 h at 40 °C. This was followed by incubation with an anti-digoxin antibody at 37 °C for 2 h. BCIP/NBT was used for colorimetric detection of circKLHL24. The sequence of the circKLHL24 probe is listed in Supplementary Table S2. The staining intensity and the proportion of positive cells were used to determine the staining scores for circKLHL24. Staining intensity was scored as: 0 (none), 1 (light blue), 2 (blue), and 3 (dark blue). The proportion of positively stained cells was scored as: 1 (< 25%), 2 (25–50%), 3 (50–75%), and 4 (75–100%). The final score was calculated by multiplying the staining intensity by the proportion of positively stained cells. Patients were then divided into high and low expression groups based on the median ISH score.

### Immunohistochemistry (IHC)

Histological sections from formalin-fixed, paraffin-embedded tissues were subjected to antigen retrieval in citrate buffer for 15 min, followed by blocking with normal goat serum for 30 min. The tissue sections were then incubated overnight at 4 °C with primary antibodies, including CXCL12, Sec31A, and S100. Avidin-biotin peroxidase detection systems with DAB substrate were used to visualize the antigen locations, followed by counterstaining with hematoxylin. Immunohistochemical signal intensity and the proportion of positively stained areas in the tissue sections were evaluated and scored independently by two observers. The staining intensity was scored as follows: 0 (no staining), 1 (light), 2 (intermediate), and 3 (strong). The proportion of positive cells was scored as: 1 (< 25%), 2 (25–50%), 3 (50–75%), and 4 (75–100%). The final score was calculated by multiplying the staining intensity score by the proportion of positively stained cells.

### RNA immunoprecipitation (RIP)

RIP experiments were performed using the EZ-Magna™ RIP Kit (Millipore, USA) following the manufacturer’s protocol. Briefly, cultured CAFs were lysed in RIP lysis buffer supplemented with protease inhibitor cocktail and RNase inhibitor. After brief sonication, the lysates were centrifuged to remove debris. A total of 100 µl of cleared lysate was incubated with 5 µg of antibody at 4 °C overnight with gentle rotation. The next day, Magna™ A/G magnetic beads were equilibrated and added to each reaction, followed by incubation at room temperature for 30–60 min. The bead–antibody–RNA–protein complexes were washed three to four times with RIP wash buffer. RNA was then eluted using the kit’s elution buffer and purified using the Trizol reagent (Invitrogen), followed by downstream qRT-PCR analysis.

### RNA pull-down assay

RNA circularization and antisense probe generation were performed as described Song et al. [18], The full-length sequence of circKLHL24 was cloned into a vector containing a T7 promoter and linearized for in vitro transcription. Transcription was carried out using the RiboMax Express Large Scale RNA Production System (Promega, P1320). Each 20 µl reaction contained 1 µg DNA template, 0.5 mM dNTPs, and 2 µl T7 RNA polymerase, and was incubated at 37 °C for 2 h. After transcription, DNase I (NEB, M0303S) treatment was performed for 15 min at 37 °C to remove template DNA. Transcribed RNAs were ethanol-precipitated and resuspended in RNase-free water. For circularization, 50 µg of transcribed linear RNA was incubated with a 20-nucleotide DNA bridge oligonucleotide and T4 RNA ligase 1 (NEB, M0204L) at 16 °C overnight in a 500 µl reaction. Circular and linear RNAs were separated by denaturing urea-PAGE, stained with ethidium bromide, visualized under UV light, and excised for gel extraction. RNA circularization was confirmed by RNase R digestion. For RNA pull-down, biotin-labeled circKLHL24 RNA probes were incubated with cell lysates, and streptavidin-coated magnetic beads were used to capture RNA–protein complexes. The protein bound to the beads was then eluted and analyzed by mass spectrometry (MS) or western blotting. Silver staining was carried out using the Pierce Silver Stain Kit (24612, Thermo Scientific, USA) according to the provided protocol.

### Golgi fractionation

The Golgi isolation kit (Sigma-Aldrich) was used to isolate endosomes following the manufacturer’s protocol. In brief, 5 × 10⁷ cells were harvested and washed with ice-cold PBS. A 0.25 M sucrose solution was then added to the cells, and the suspension was homogenized and centrifuged. Subsequently, a 2.3 M sucrose solution was added to achieve a final sucrose concentration of 1.25 M. A discontinuous gradient was formed in an ultracentrifuge tube, and the tubes were centrifuged at 120,000 × g for 3 h at 4 °C. The Golgi-enriched fraction was then collected from the interphase between the 1.1 M and 0.25 M sucrose layers.

### ER fractionation

ER enrichment was performed using the Minute ER Enrichment Kit (Invent Biotechnologies, ER-036). In brief, cells or tissues were first frozen at − 80 °C, then suspended or ground in buffer A. After centrifugation, the supernatants were processed with the specified buffers as per the kit instructions. Finally, the ER-enriched pellets were resuspended in 1× SDS loading buffer and analyzed by immunoblotting for the target proteins.

### Luciferase reporter assay

The wild-type or mutant circKLHL24 plasmids and miR-615-5p mimic were co-transfected into CAFs. After transfection, the cells were seeded in 96-well plates, and luciferase activity was measured using the Dual-Luciferase Reporter Assay System (Promega, USA) following the manufacturer’s guidelines.

### Animal experiments

In the murine sciatic nerve invasion model, 4-week-old BALB/c nude mice (*n* = 10) were anesthetized, and their sciatic nerves were surgically exposed. A mixture of tumor cells (1 × 10⁴ Miacapa2 cells) and primary CAFs (3 × 10⁴) was injected into the right sciatic nerve under the perineurium using a custom Hamilton syringe, with 2 µL of PBS. Sciatic nerve function was assessed weekly on a scale from 4 (normal) to 1 (totally paralyzed paws). The sciatic nerve index was measured biweekly by calculating the distance between the first and fifth toes of the hind limbs. After 6 weeks, all mice were sacrificed, and the sciatic nerve and tumor tissues were harvested and fixed for histological analysis.

### For the genetically engineered mouse model

KPC (LSL-KRAS G12D/+; LSL-TP53 R172H/+; PDX-1-CRE+/+) mice were purchased from Shanghai Model Organisms. Mice were randomly assigned into three groups. Mice were injected with AAV-packaged shNC (AAV-shNC) ( *n* = 10) or with AAV-packaged shcircKLHL24 (AAV-shcircKLHL24) ( *n* = 10) into the tail vein at a dose of 7 × 10^11^ viral genomes (vg) per mouse. The third group will receive an intraperitoneal injection of CXCL12 neutralizing antibody at a dose of 40 mg/kg, once every three days. After 4 weeks of treatment, all mice were sacrificed, and the pancreas was harvested, fixed, and processed for histological analysis.

### RNA-seq

Total RNA was isolated and purified using TRIzol (Life, USA) following the manufacturer’s procedure. After the quality inspection of Agilent 2100 Bioanalyzer (Agilent, USA) and NanoPhotometer (Implen, Germany), ribosomal RNA was removed from 1 µg total RNA. VAHTS Universal V6 RNA-seq Library Prep Kit for Illumina (Vazyme, China) was used for circRNA library construction following the manufacturer’s protocol. Each library was sequenced on an Illumina Novaseq 6000 (Illumina Corporation, USA) in 150PE mode following the vendor’s recommended protocol by Guangzhou Huayin Health Medical Group CO.,Ltd. (Guangzhou, China).

### Statistical analysis

All experimental data were presented as mean ± standard deviation (SD) using GraphPad Prism 9.0. Differences between parametric variables were assessed using Student’s t-test or one-way analysis of variance (ANOVA), while nonparametric variables were analyzed using the Mann-Whitney U test. Survival analysis was conducted using Kaplan-Meier curves and the log-rank test. Pearson’s two-sided correlation was used for correlation analysis. Multivariate analysis of relative risk was performed using Cox regression. A *p*-value of < 0.05 was considered statistically significant.

## Results

### circKLHL24 correlates with perineural invasion and poor prognosis in pancreatic cancer

**Fig. 1:**
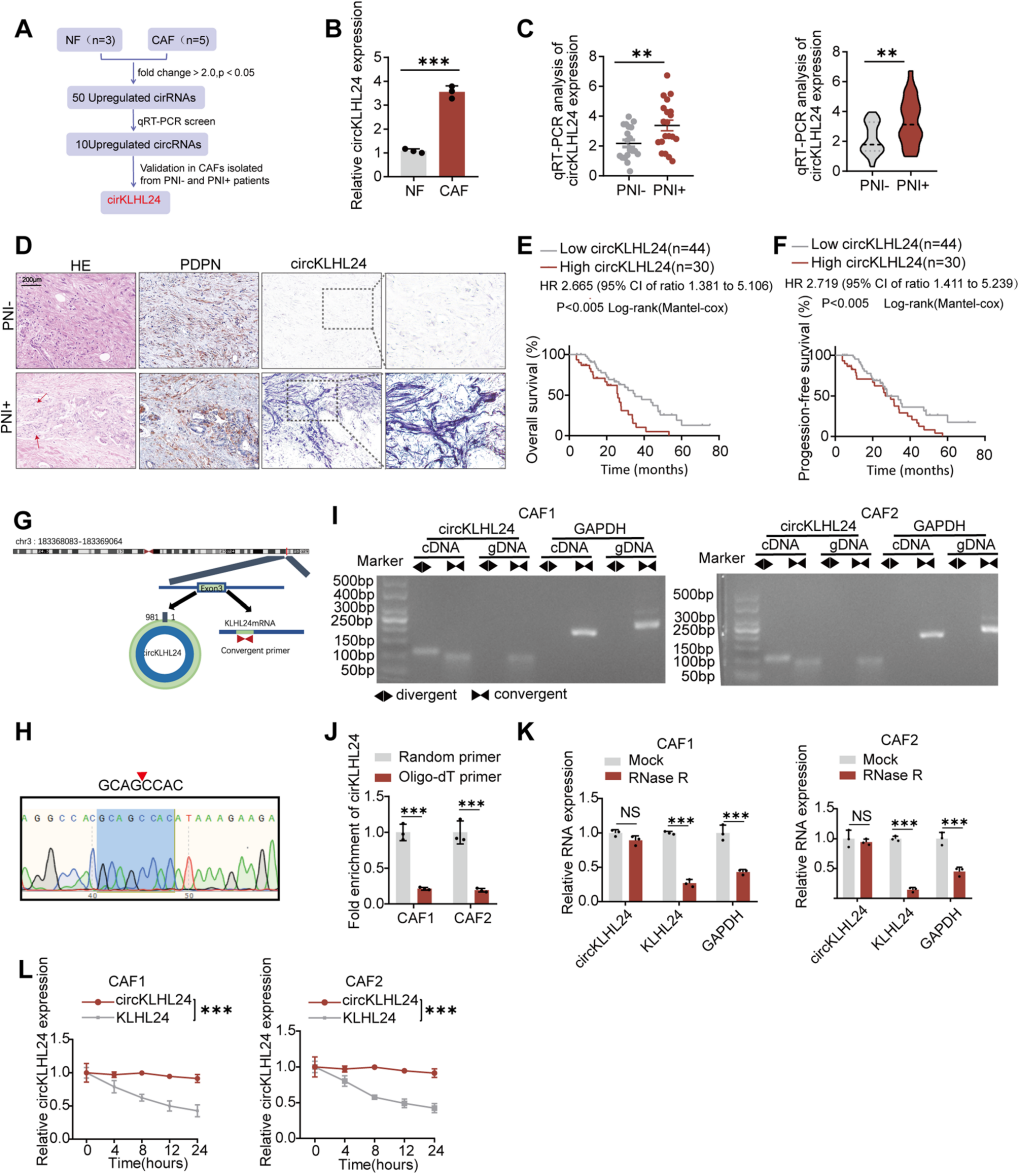
circKLHL24 Correlates with Perineural Invasion and Poor Prognosis in Pancreatic Cancer. (**A**) Schematic illustration of the identification of circKLHL24 upregulated in CAFs. (**B**) qRT–PCR analysis of circKLHL24 expression in NFs and CAFs (**C**) Quantification of circKLHL24 expression by using qRT–PCR in PNI (*n* = 20) and non-PNI (*n* = 20) PDAC tissues. The left panel shows the plot of circKLHL24 expression in each tissue. Right panel shows the expression as violin plots. (**D**) Representative images of H&E staining, PDPN immunohistochemistry (CAF marker), and circKLHL24 in situ hybridization (ISH) in PNI-positive (PNI+, *n* = 43) and PNI-negative (PNI−, *n* = 31) pancreatic ductal adenocarcinoma (PDAC) tissues. Red arrows indicate representative perineural invasion in H&E staining. Scale bar, 200 μm. (**E**‑**F**) Kaplan–Meier survival curves of overall survival and progression-free survival for PDAC patients with high (*n* = 44) or low circKLHL24 expression(*n* = 30) evaluated by ISH. A univariate Cox regression model was used to calculate the hazard ratio (HR). (**G**) Schematic illustration showing the genomic loci of circKLHL24. circKLHL24 was generated by exons 3 of KLHL24. (**H**) The back-splice junction of circKLHL24 was identified by Sanger sequencing. (**I**) cDNA and gDNA of CAF1 and CAF2 were amplified with convergent and divergent primers. GAPDH was as the negative control. (**J**) Fold enrichment of circKLHL24 with oligo-dT primers or random primers. PCR analysis of circKLHL24, KLHL24, and GAPDH expression in CAF1 and CAF2 cells treated with or without RNase R. qRT–PCR analysis of circKLHL24 and KLHL24 mRNA in CAF1 and CAF2 cells treated with actinomycin D at the indicated time points. n.s. *= no significance*, **P < 0.01, ***P < 0.001

To elucidate the pivotal circRNAs involved in the interplay between cancer-associated fibroblasts (CAFs) and tumor cells during perineural invasion (PNI), we conducted a high-throughput sequencing analysis (GSE172096) on CAFs and their corresponding adjacent normal fibroblasts (NFs) (Fig. 1A). Subsequent quantitative real-time polymerase chain reaction (qRT-PCR) validation revealed that, among 50 upregulated circRNAs (with a fold-change > 2 and *P* < 0.05), circKLHL24 exhibited a significant elevation in CAFs (Fig. 1A-B). Notably, circKLHL24 expression was markedly higher in patients with severe PNI (PNI+) compared to those without severe PNI (PNI-) (Fig. 1C). Serial staining of adjacent PDAC tissue sections using H&E, PDPN immunohistochemistry (a CAF marker), and ISH revealed a high degree of spatial co-localization between PDPN-positive stromal areas and circKLHL24 signal in patients with severe PNI (Fig. 1D). ImageJ-based quantification of circKLHL24 ISH signals in 74 PDAC tissues (43 PNI⁺, 31 PNI⁻) demonstrated significantly higher expression in PNI⁺ cases (Figure S4A). Additionally, an increased expression of circKLHL24 was significantly correlated with reduced overall survival (OS) and progression-free survival (PFS) in a multicenter cohort (Fig. 1E-F).

To ascertain the origin of circKLHL24, we consulted the UCSC Genome Browser and discovered that it originated from exon 3 of the KLHL24 gene (chr3: 183368083–183369064) (Fig. 1G). Utilizing Sanger sequencing and nucleic acid electrophoresis assays, we verified the presence of circKLHL24, which was amplified exclusively from cDNA, but not from gDNA (Fig. 1H-I). Moreover, circKLHL24 exhibited a greater challenge to reverse transcribe with oligo-dT primers compared to random primers, suggesting the absence of a poly-A tail (Fig. 1J). In line with expectations, circKLHL24 demonstrated heightened resistance to RNase-R digestion and possessed a longer half-life than its parent linear transcript in CAFs upon treatment with RNase R and actinomycin D (Fig. 1K-L). These findings imply that circKLHL24 possesses a covalently closed continuous loop structure and is associated with PNI and poor prognosis in pancreatic cancer.

### circKLHL24 strengthens the capacity of cafs to drive perineural invasion in pancreatic cancer

**Fig. 2:**
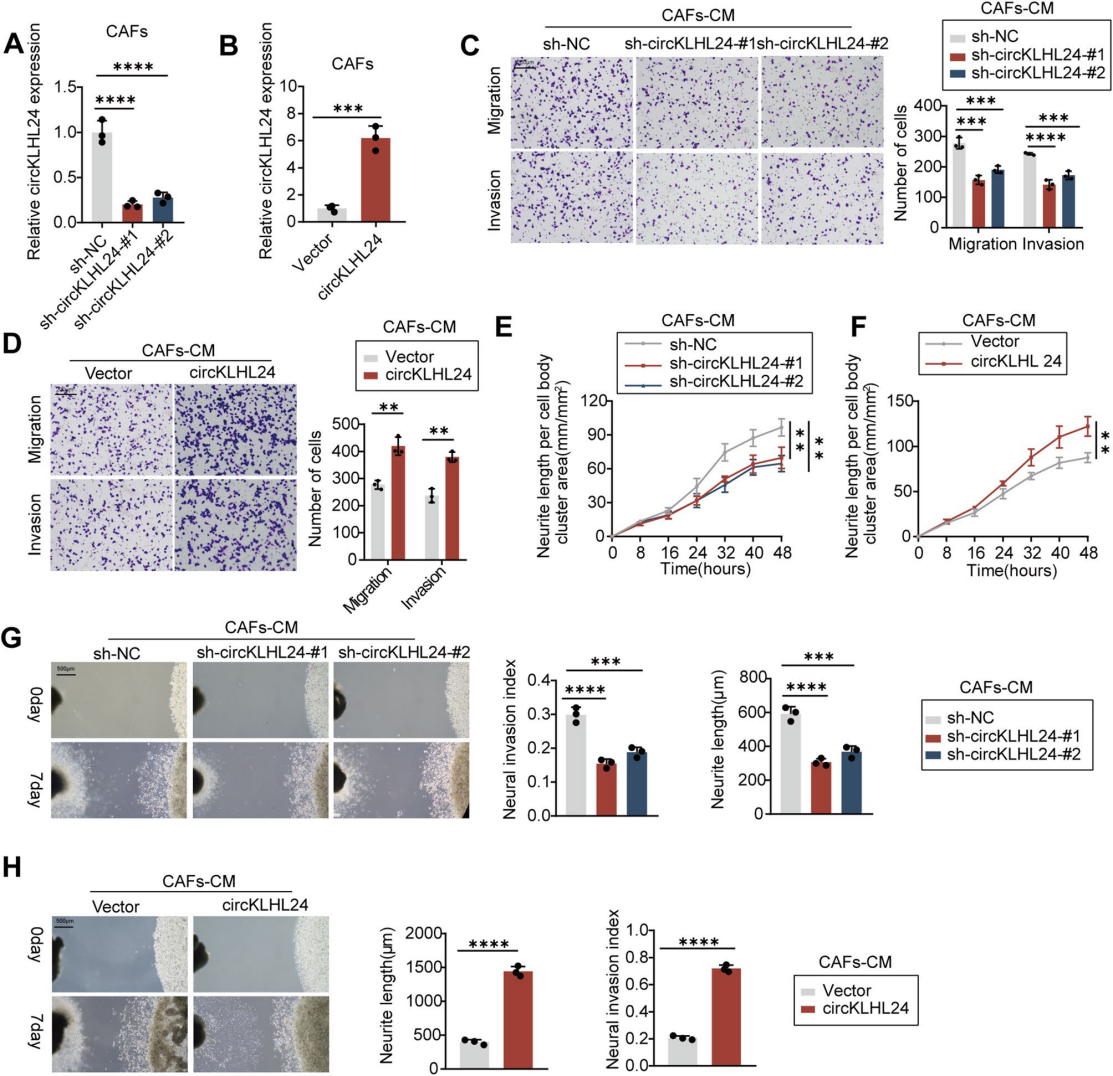
circKLHL24 is critical for CAFs to drive perineural invasion in PDAC cells. Miapaca2 cells and murine DRG were grown in conditioned medium (CM) from CAFs stably transfected with empty vector, circKLHL24, lenti-NC-shRNA or lenti-circ KLHL24-shRNA for 2 weeks and then subjected to the indicated experiments. (**A-B**) qRT–PCR analysis of circKLHL24 expression in CAFs. (**C-D**) Representative image (left panel) and quantitative analysis (right panel) of Miapaca2 cell migration and invasion in the Transwell assay. Scale bars, 200 μm. (**E-F**) Quantitative analysis of murine DRG neurite length per cell body cluster area. (**G‑H**) Representative image (left panel) of a co-culture system involving DRG neurons and Miapaca2 cells. Quantitative analysis (right panel) assessing the neuroinvasive potential of Miapaca2 cells. Scale bar, 500 μm. **P < 0.01, ***P < 0.001, ****P < 0.0001

To explore the effect of circKLHL24 on PNI of pancreatic cancer in vitro, circKLHL24 was depleted in CAFs via lenti-circKLHL24-shRNA that targeted the back-spliced region of circKLHL24 or upregulated via circKLHL24-overexpressing plasmid (Fig. 2A-B). Transwell assays showed that circKLHL24 knockdown in CAFs dramatically abolished their ability to induce migration and invasion of MiaPaCa-2, while circKLHL24 overexpression facilitated CAFs to drive tumor cells migration and invasion (Fig. 2C and D). In addition, we cocultured CAFs with neuron cells for 1 week. The results showed that circKLHL24 knockdown in CAFs dramatically abolished their ability to promote neurite growth of neuron cells, while circKLHL24 overexpression facilitated CAFs to drive neuron cells neurite growth (Fig. 2E and F). Consistent with these findings, a 3D coculture model of tumor cells and dorsal root ganglion (DRG) further confirmed that circKLHL24 knockdown in CAFs dramatically abolished their ability to drive tumor cells invade the DRG and promote DRG neurite outgrowth (Fig. 2G and H).

### circKLHL24 promotes cxcl12 expression and secretion in cafs to drive perineural invasion

**Fig. 3:**
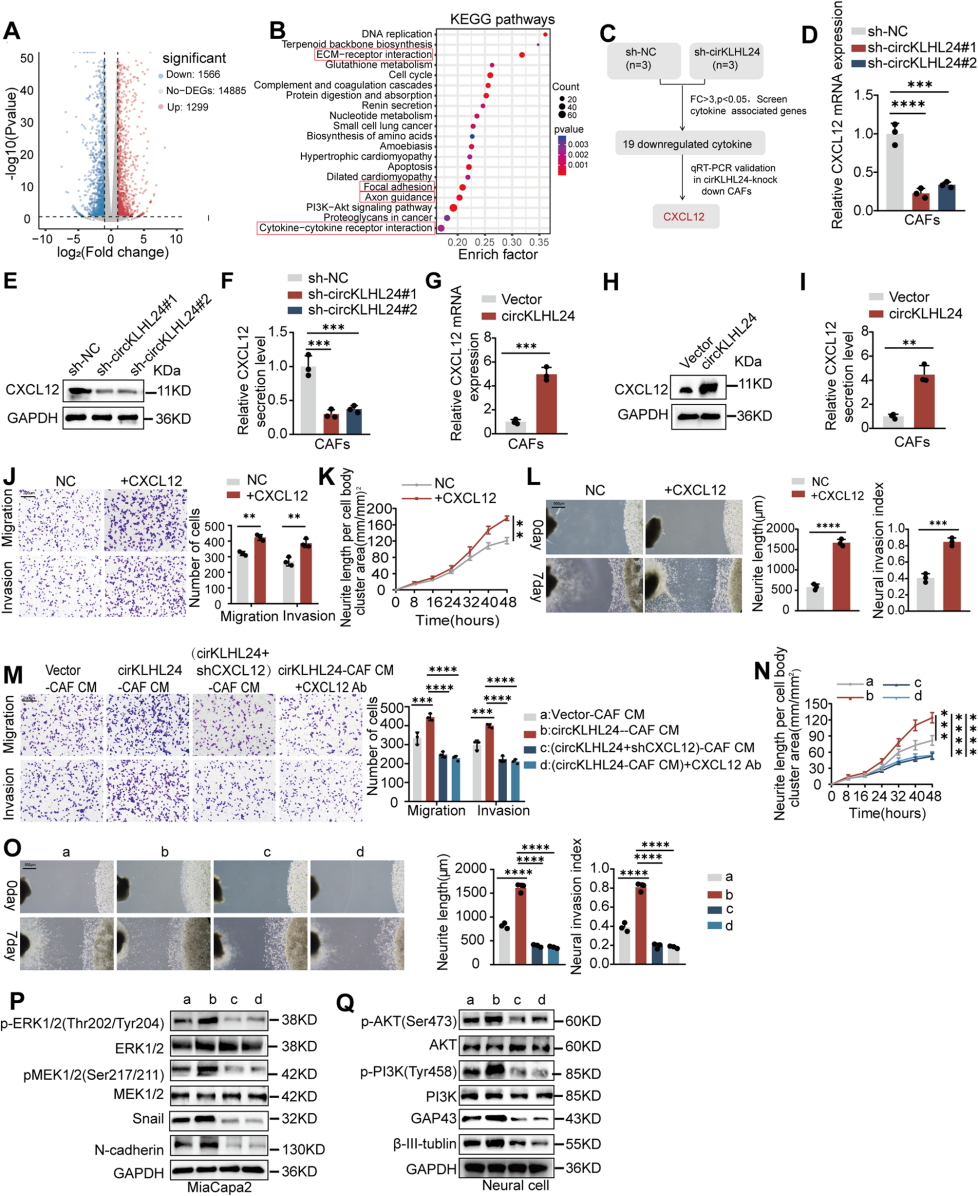
circKLHL24 promotes CXCL12 expression and secretion in CAFs. (**A**) Plot showing the sums of the expression levels of genes regulated by circKLHL24. (**B**) Top 20 enriched pathways of differential mRNA expression between CAFs and circKLHL24 knock down CAFs. (**C**) Flow chart for the identification of CXCL12 as the downstream target of circKLHL24. (**D‑I**) The mRNA level (**D**, **G**), protein level (**E**, **H**), and secretion level (**F**, **I**) of CXCL12 in CAFs transfected with vector, circKLHL24, sh-NC, or sh-circKLHL24. (J-L) Miapaca2 and DRG were grown in DMEM with or without exogenous supplementation of CXCL12. (**J**) Representative image (left panel) and quantitative analysis (right panel) of Miapaca2 migration and invasion in the Transwell assay. Scale bars, 200 μm. (**K**) Quantitative analysis of DRG neurite length per cell body cluster area. (**L**) Representative image (left panel) of a co-culture system involving DRG and Miapaca2. Quantitative analysis (right panel) assessing the neuroinvasive potential of Miapaca2. Scale bar, 500 μm. (**M-Q**) Miapaca2 and DRG were grown in conditioned medium (CM) from CAFs transfected with vector or circKLHL24, Lenti-CXCL12 shRNA or treated with a neutralizing antibody against CXCL12 (120ug/ml). (**M**) Representative image (left panel) and quantitative analysis (right panel) of Miapaca2 migration and invasion in the transwell assay with the indicated treatment. Scale bars, 200 μm. (**N**) Quantitative analysis of DRG neurite length per cell body cluster area with the indicated treatment. (**O**) Representative image (left panel) of a co-culture system involving DRG and Miapaca2. Quantitative analysis (right panel) assessing the neuroinvasive potential of Miapaca2, with the indicated treatment. Scale bar, 500 μm. (**P**) Western blot of MAPK/ERK signaling pathway, Snail and N-cadherin in Miapaca2 with the indicated treatment. (**Q**) Western blot of PI3K/Akt pathway, GAP43 and β-III tubulin in neuronal cells with the indicated treatment. **P < 0.01, ***P < 0.001, ****P < 0.0001

To investigate the functional role of circKLHL24 in CAFs, we performed next-generation sequencing to compare gene expression profiles following circKLHL24 knockdown in CAFs (Fig. 3A). Kyoto Encyclopedia of Genes and Genomes (KEGG) pathway analysis revealed significant suppression of cytokine-related signaling pathways in circKLHL24-knockdown CAFs, suggesting a potential role of circKLHL24 in regulating tumor-stroma interactions (Fig. 3B). Further analysis identified 19 cytokine-related genes that were downregulated by at least three-fold upon circKLHL24 knockdown, among which CXCL12 exhibited the most significant reduction, as confirmed by qPCR (Fig. 3C). Q-PCR, Western blot and ELISA assays further confirmed a significant decrease in both CXCL12 expression and secretion in CAFs upon circKLHL24 knockdown (Figs. 3D-F), whereas circKLHL24 overexpression led to a marked increase in CXCL12 levels (Fig. 3G-I). Exogenous CXCL12 supplementation significantly enhanced tumor cell migration and invasion while also promoting neurite outgrowth in neuronal cells (Fig. 3J-L). Consistently, CXCL12 silencing or neutralization in circKLHL24-overexpressing CAFs significantly attenuated their ability to promote tumor cell migration and invasion, as well as their capacity to stimulate neurite outgrowth (Fig. 3M-N). In a 3D co-culture system comprising tumor cells and DRG, CXCL12 silencing or neutralization in circKLHL24-overexpressing CAFs completely abolished their ability to promote tumor cell invasion into the DRG and enhance DRG neurite outgrowth (Fig. 3O). Mechanistically, Western blot analysis showed that CAF-derived CXCL12 activated MAPK/ERK signaling pathway in pancreatic cancer cells, thereby upregulating the upregulation of EMT-related genes (Snail and N-cadherin) (Fig. 3P). In neuronal cells, CXCL12 activated the PI3K/Akt pathway, promoting the expression of neurogenesis-associated genes (GAP43 and β-III tubulin) (Fig. 3Q). Together, these findings suggest that circKLHL24 enhances the pro-tumorigenic and pro-neurogenic activity of CAFs via upregulation of CXCL12 expression and secretion.

### circKLHL24 interacts with sec31a and prevents its degradation

**Fig. 4:**
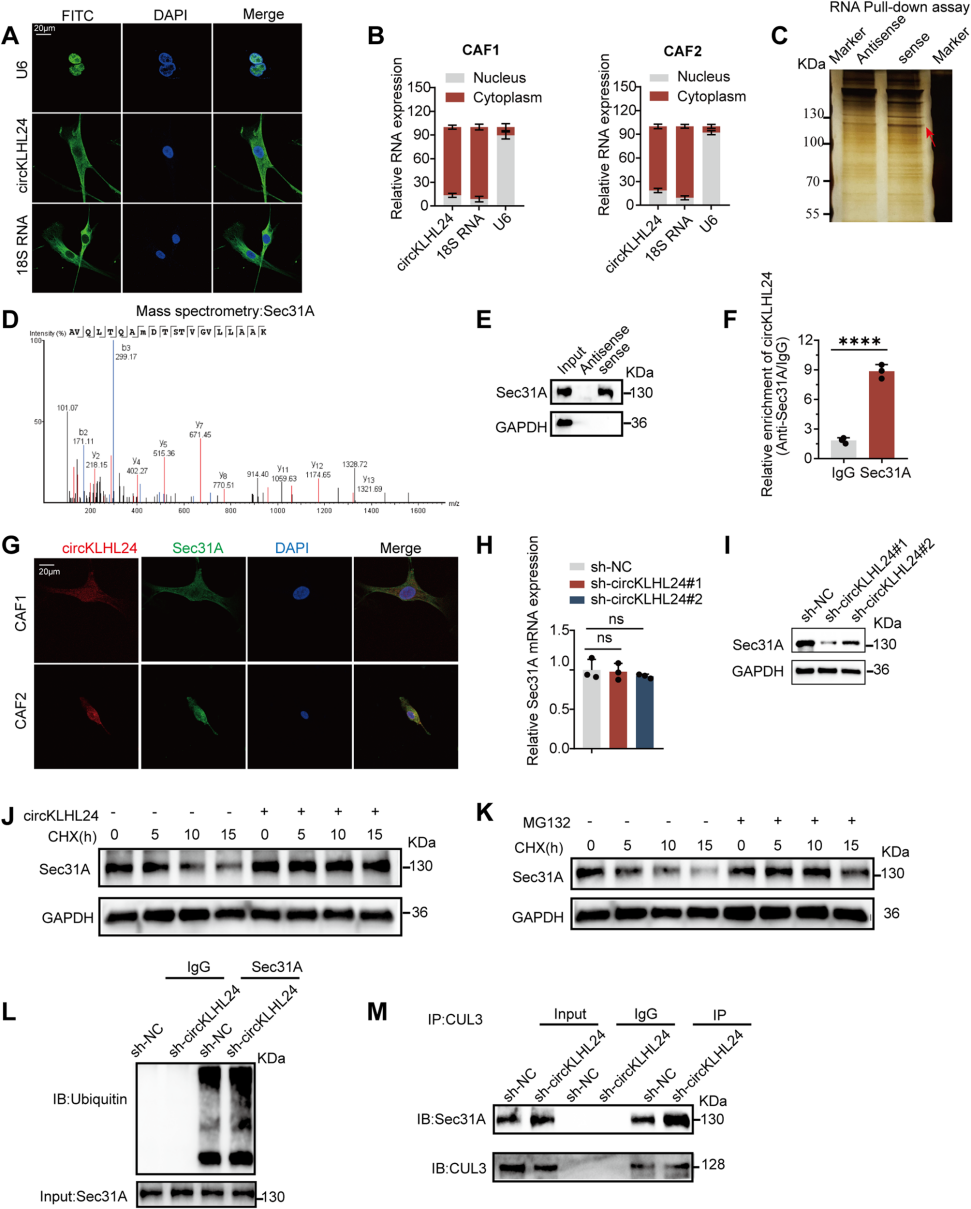
circKLHL24 interacts with Sec31A and prevents its degradation. (**A**) Representative RNA-FISH images showing the subcellular localization of circKLHL24 in CAF. U6 (nuclear marker) and 18 S rRNA (cytoplasmic marker) were used as internal controls. Scale bars, 20 μm. (**B**) Subcellular fractionation assays of circKLHL24 in CAF1 and CAF2. Data are expressed as the mean ± SD. (**C**) Silver staining for RNA pull-down assay with the specific biotin-labeled circKLHL24 probe in CAF lysates. Red arrows indicate the unique differential band precipitated by the circKLHL24 probe. (D-E) Mass spectrometry (**D**) and western blot (**E**) analysis of proteins in unique differential bands. Sec31A was identified as a candidate protein interacting with circKLHL24. (**F**) RNA immunoprecipitation (RIP) assays in CAFs using IgG and Sec31A antibodies. The relative enrichment of circKLHL24 was calculated by qRT–PCR. (**G**) Dual RNA-FISH staining assay indicating the colocalization of circKLHL24 (red) and Sec31A (green), with nuclear staining with DAPI (blue). Scale bars, 20 μm. (**H-I**) The mRNA and protein levels of Sec31A in CAFs transfected with sh-NC or sh- circKLHL24. (**J**) Western blot of the expression kinetics of Sec31A in CAFs transfected with empty vector or circFARP1 plasmid and treated with CHX (100 µg/ml) for 0 h, 5 h, 10 h, or 15 h (GAPDH as a control). (**K**) Western blot of the expression kinetics of Sec31A in CAFs treated with or without MG132 (20 µM) after CHX (100 µg/ml) treatment for 0 h, 5 h, 10 h, and 15 h (GAPDH as a control). (**L**) Immunoprecipitation of Sec31A protein in lysates from CAFs with or without circKLHL24 silencing, followed by immunoblotting with an anti-ubiquitin antibody. MG132 (20 µM) was added before cell lysis to inhibit Sec31A degradation. (**M**) Immunoprecipitation assays demonstrating the interaction of UPS8 and Sec31A in CAFs with the indicated treatments. Data are expressed as the mean ± SD. ****P < 0.0001

**Fig. 5:**
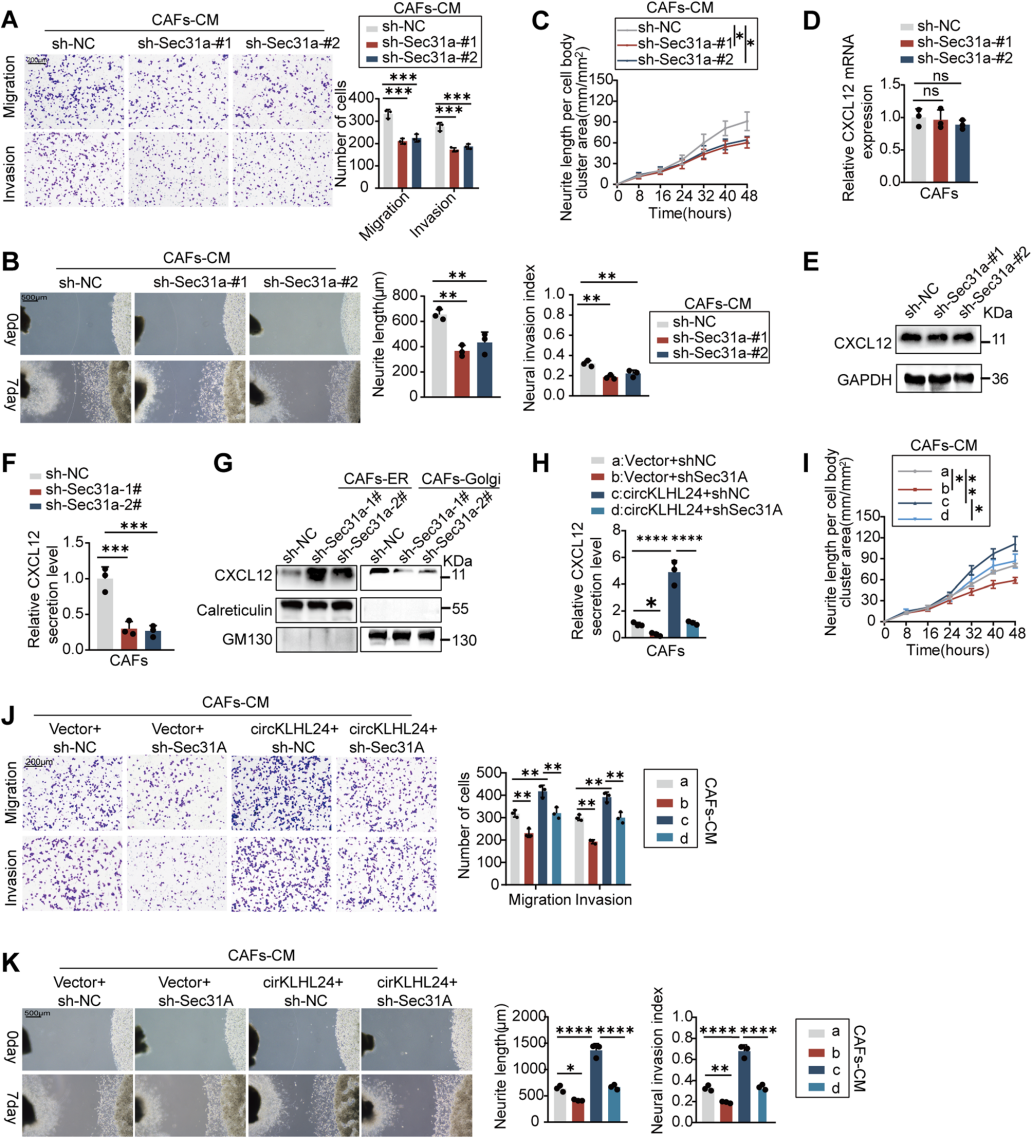
circKLHL24 facilitates CXCL12 secretion by regulating Sec31A-mediated trafficking. (**A‑C**) Miapaca2 and DRG cells were grown in conditioned medium (CM) from CAFs transfected with lenti-NC shRNA, lenti-Sec31A shRNA, vector or circKLHL24. (**A**) Representative image (left panel) and quantitative analysis (right panel) of Miapaca2 cell migration and invasion in the Transwell assay. Scale bars, 200 μm. (**B**) Representative image (left panel) of a co-culture system involving DRG neurons and Miapaca2 cells. Quantitative analysis (right panel) assessing the neuroinvasive potential of Miapaca2 cells. Scale bar, 500 μm. (**C**) Quantitative analysis of DRG neurite length per cell body cluster area. (**D-F**) The mRNA level (**D**), protein level (**E**), and secretion level (**F**) of CXCL12 in CAFs transfected with Lenti-NC shRNA or Lenti-Sec31A shRNA. (**G**) Western blot analysis of CXCL12 in ER or Golgi of CAFs transfected with Lenti-NC shRNA or Lenti-Sec31A shRNA. (**H-K**) Miapaca2 and DRG cells were grown in conditioned medium (CM) from CAFs transfected with vector or circKLHL24 or lenti-NC shRNA or Lenti-Sec31A shRNA. (**H**) The secretion level of CXCL12 in CAFs transfected with the indicated treatment. (**I**) Quantitative analysis of DRG neurite length per cell body cluster area with the indicated treatment. (**J**) Representative image (left panel) and quantitative analysis (right panel) of Miapaca2 cell migration and invasion in the transwell assay with the indicated treatment. Scale bars, 200 μm. (**K**) Representative image (left panel) of a co-culture system involving DRG neurons and Miapaca2 cells. Quantitative analysis (right panel) assessing the neuroinvasive potential of Miapaca2 cells with the indicated treatment. Scale bar, 500 μm. *P < 0.05, **P < 0.01, ***P < 0.001, ****P < 0.0001

To elucidate how circKLHL24 regulates CXCL12 expression and secretion, fluorescence in situ hybridization (FISH) and subcellular fractionation assays were performed, confirming its localization in both cytoplasmic and nuclear compartments of CAFs (Fig. 4A-B). Given its cytoplasmic presence, potential interacting proteins were investigated using an RNA pulldown assay with biotinylated circKLHL24 probes targeting the back-spliced junction. GO enrichment analysis of proteins dysregulated upon circKLHL24 knockdown revealed significant enrichment in the endoplasmic reticulum lumen category (Figure S4D), suggesting possible ER-related protein interactors. Based on this and silver staining of the 130 kDa region from RNA pulldown assays (Fig. 4C), we shortlisted Sec31A, COPβ, and COPG for further investigation. Mass spectrometry identified Sec31A with high confidence, supported by 42 unique peptides (Fig. 4D, FigureS5, S6, S7, S8, S9, Supplemental Table S4). We validated these candidates via RNA pulldown followed by western blotting, which showed that only Sec31A was enriched by the sense circKLHL24 probe (Fig. 4E), whereas COPβ, and COPG were not detected (Fig. 4E-F). To confirm specificity, we further performed RIP-qPCR using antibodies against Sec31A, COPβ, and COPG. Again, only the Sec31A antibody significantly enriched circKLHL24, whereas COPβ, and COPG did not (Fig. 4F, Figure S4G–H), indicating a specific and reproducible interaction. To further verify the functional specificity of this interaction, we performed truncation analysis and mapped the Sec31A-binding region of circKLHL24 to the 1–100 nt fragment. Notably, site-directed mutation within this region abolished the ability of circKLHL24 to pull down Sec31A, as demonstrated by RNA pulldown assays (Figure S10A–B). FISH-immunofluorescence (IF) co-localization assays confirmed the cytoplasmic co-localization between circKLHL24 and Sec31A (Fig. 4G). Despite no detectable changes in Sec31A mRNA levels upon circKLHL24 depletion, Sec31A protein levels were significantly reduced, suggesting a post-transcriptional regulatory mechanism (Fig. 4H-I). A cycloheximide (CHX) chase assay revealed accelerated Sec31A degradation following circKLHL24 knockdown, which was effectively blocked by MG132 treatment, implicating the ubiquitin-proteasome pathway in this process (Fig. 4J-K). Sec31A ubiquitination was markedly increased in circKLHL24-knockdown cells (Fig. 4L). Previous studies reported that CUL3 facilitates Sec31A degradation. Co-immunoprecipitation (Co-IP) assays demonstrated that circKLHL24 interferes with the Sec31A-CUL3 interaction, stabilizing Sec31A protein levels (Fig. 4M). These findings indicate that circKLHL24 binds directly to Sec31A and prevents its ubiquitin-mediated degradation.

### circKLHL24 facilitates cxcl12 secretion by regulating sec31A-mediated trafficking

Silencing Sec31A in CAFs impaired their ability to promote pancreatic cancer cell migration, invasion, and neural cell neurite outgrowth, closely resembling the effects observed with circKLHL24 depletion (Fig. 5A and C). Interestingly, Sec31A depletion did not affect CXCL12 mRNA or intracellular protein levels, but significantly reduced its secretion, as revealed by ELISA assays (Fig. 5D and F). Sec31A is a key component of the COPII transport system, mediating ER-to-Golgi trafficking essential for cytokine secretion [19]. To further investigate whether Sec31A influences CXCL12 transport along the secretory pathway, ER and Golgi compartments were fractionated to assess CXCL12 distribution by Western blotting. CXCL12 accumulated in the ER and was markedly reduced in the Golgi upon Sec31A knockdown, indicating that Sec31A is essential for efficient ER-to-Golgi trafficking of CXCL12 (Fig. 5G). Furthermore, the circKLHL24-induced increase in CXCL12 secretion was largely abolished upon Sec31A depletion. (Fig. 5H). Functionally, the knock down of Sec31A significantly counteracted the effects of circKLHL24 overexpression, leading to a marked reduction in pancreatic cancer cell migration and invasion, as well as impaired neurite outgrowth in neural cells (Fig. 5I and K). Together, these findings demonstrate that circKLHL24 enhances CXCL12 secretion through Sec31A-mediated ER-to-Golgi trafficking.

### circKLHL24 upregulates CXCL12 expression by sponging miR-615-5p in CAFs

**Fig. 6:**
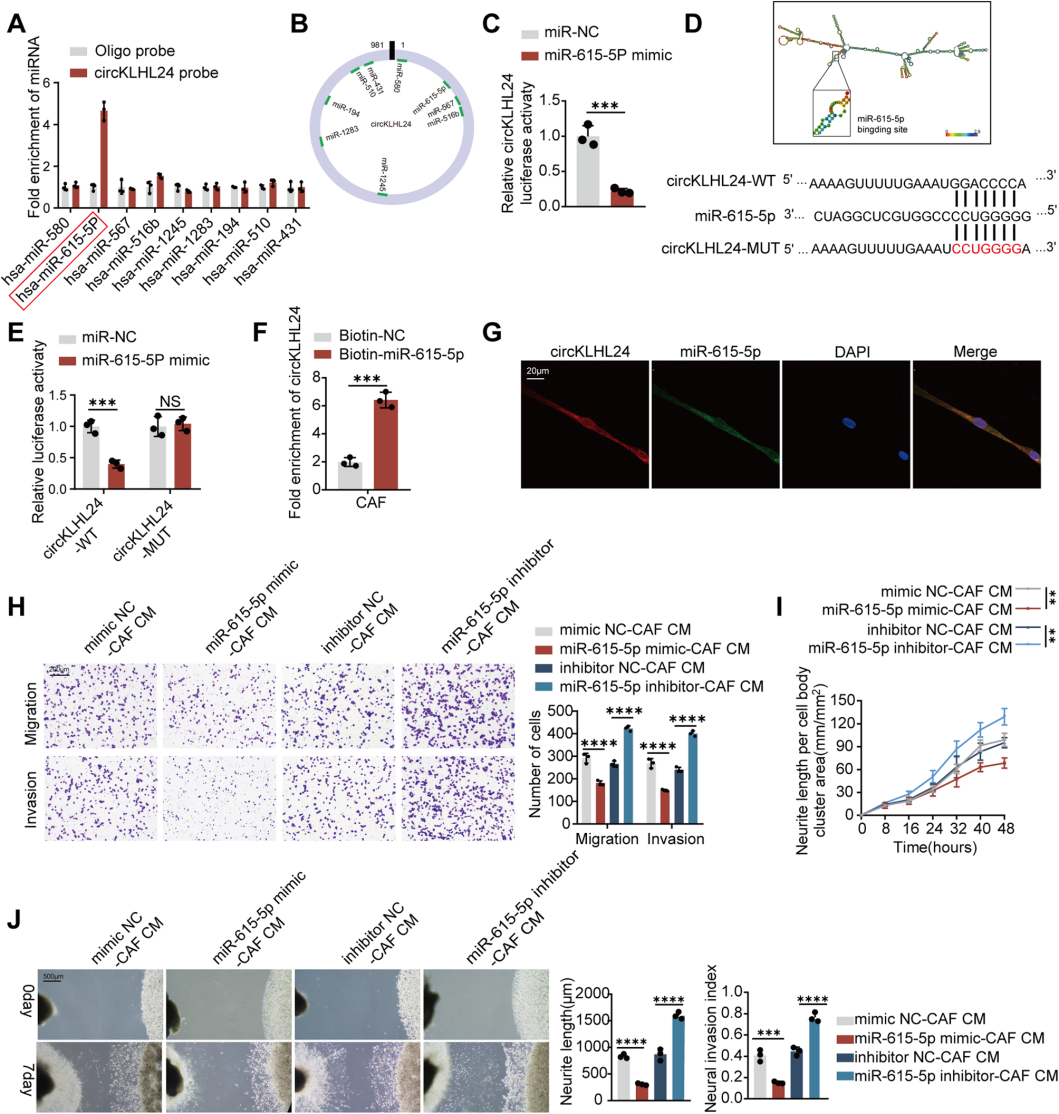
circKLHL24 functions as a miR-615-5p sponge in CAFs. (**A-B**) qRT–PCR analysis of the enrichment of the indicated miRNAs in CAFs by RNA pull-down assay. (**C**) The luciferase activities of the circKLHL24 luciferase reporter plasmid following transfection with NC mimic or miR-615-5p mimics into CAFs. (**D**) Schematic illustration showing the sequence alignment of circKLHL24 with miR-615-5p. (**E**) The luciferase activities of the circKLHL24 luciferase reporter plasmid (WT or MUT) following transfection with NC mimic or miR-615-5p mimic into CAFs. (**F**) The fold enrichment of the circKLHL24 after pull-down assay by Biotin-NC or Biotin-miR-615-5p. (**G**) Dual RNA-FISH staining assay indicating the colocalization of circKLHL24 (red) and miR-615-5p (green), with nuclear staining with DAPI (blue). Scale bars, 20 μm. (**H-J**) Miapaca2 and DRG cells were grown in conditioned medium (CM) from CAFs transfected with NC mimic, miR-615-5p mimic, NC inhibition or miR-615-5p inhibition. (**H**) Representative image (left panel) and quantitative analysis (right panel) of Miapaca2 cell migration and invasion in the transwell assay with the indicated treatment. Scale bars, 200 μm. (**I**) Quantitative analysis of DRG neurite length per cell body cluster area with the indicated treatment. (**J**) Representative image (left panel) of a co-culture system involving DRG neurons and Miapaca2 cells. Quantitative analysis (right panel) assessing the neuroinvasive potential of Miapaca2 cells with the indicated treatment. Scale bar, 500 μm. ***P < 0.001, ****P < 0.0001

Since circKLHL24 enhances both the expression and secretion of CXCL12, we explored additional regulatory mechanisms underlying this effect. Given that circRNAs often act as cytoplasmic competing endogenous RNAs (ceRNAs), modulating post-transcriptional gene expression, we hypothesized that circKLHL24 may act as a miRNA sponge in CAFs. To identify potential miRNA interactions, bioinformatics analysis using CircInteractome predicted nine candidate miRNAs capable of binding circKLHL24 (Fig. 6A-B). Among them, miR-615-5p exhibited the strongest enrichment in circKLHL24 pull-down assays performed in CAFs, suggesting a direct interaction (Fig. 6A). Luciferase reporter assays further validated this interaction, showing that miR-615-5p overexpression significantly reduced the luciferase activity of circKLHL24 (Fig. 6C). To confirm the specificity of this binding, we performed site-directed mutagenesis of the predicted miR-615-5p binding sites on circKLHL24 (Fig. 6D). Notably, the mutant construct completely abolished the inhibitory effect of miR-615-5p on luciferase activity (Fig. 6E). Pull-down assays using biotin-labeled miR-615-5p probes demonstrated that circKLHL24 was efficiently captured by miR-615-5p, confirming their direct association in CAFs (Fig. 6F). In addition, FISH assays showed co-localization of circKLHL24 and miR-615-5p in the cytoplasm (Fig. 6G). Conditioned media from miR-615-5p-silenced CAFs significantly enhanced the migration and invasion of MiaPaCa-2 cells, as well as neurite outgrowth in neural cells (Figure H-J)). In contrast, miR-615-5p overexpression in CAFs markedly diminished these effects in MiaPaCa-2 cells (Figure H-J).

**Fig. 7:**
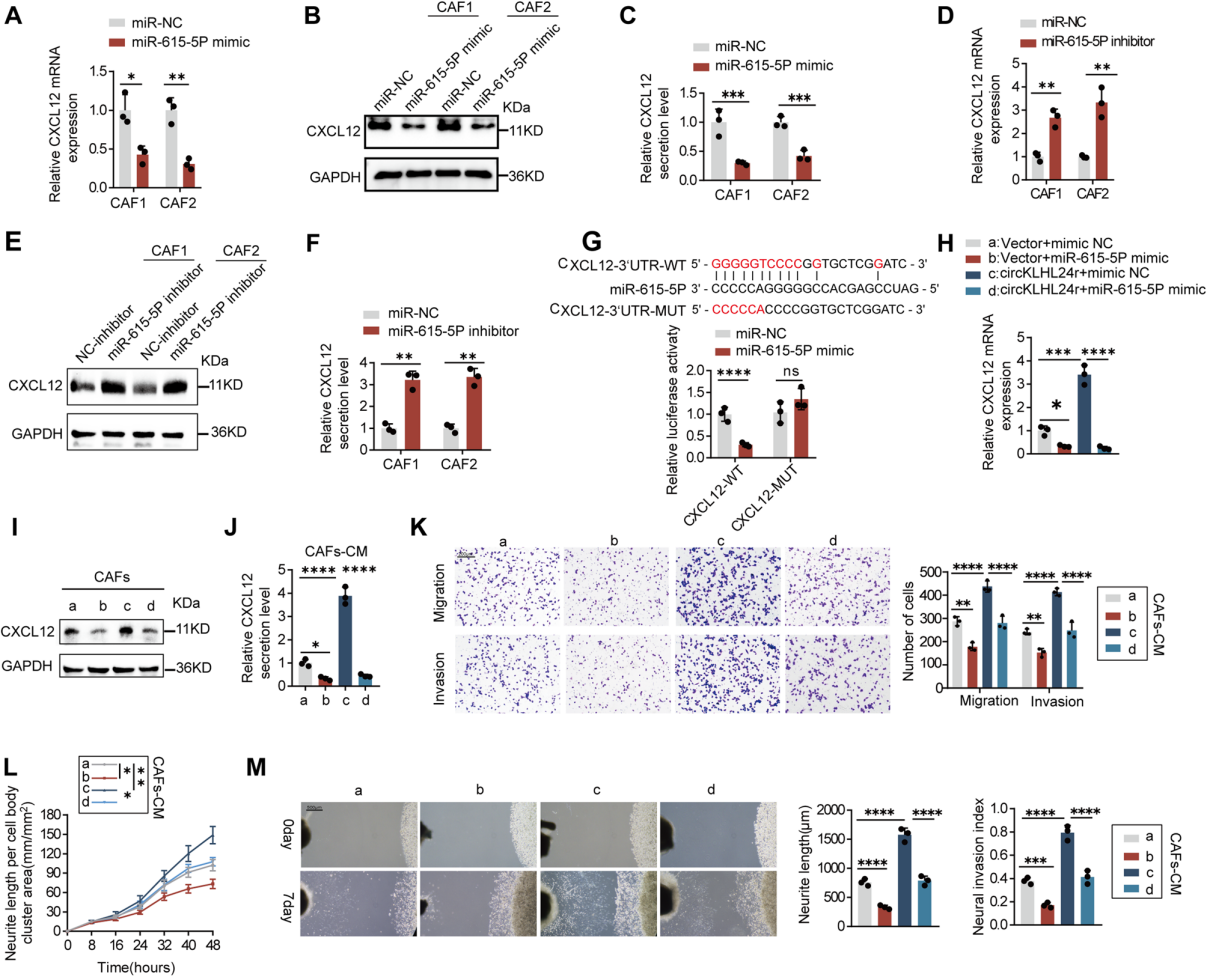
circKLHL24 enhances CXCL12 expression via miR-615-5p. (**A‑F**) The mRNA level (**A**, **D**), protein level (**B**, **E**), and secretion level (**C**, **F**) of CXCL12 in CAFs transfected with miR-NC, miR-615-5p, NC inhibition or miR-615-5p inhibition. Schematic illustrating the sequence alignment of miR-615-5p with the 3’UTR of LIF (top panel). The luciferase activities of the LIF-3’ UTR luciferase reporter plasmid containing wild-type (WT) and miR-615-5p binding site mutated (Mut) and transfected with the NC mimic or miR-615-5p mimic in CAFs (bottom panel). (**H-J**) The mRNA level (**H**), protein level (**I**), and secretion level (**J**) of CXCL12 in CAFs transfected with vector or circKLHL24. miR-NC or miR-615-5p was used to transfect in CAFs. (**K-M**) Miapaca2 and murine DRG cells were grown in conditioned medium (CM) from CAFs transfected with vector or circKLHL24. miR-NC or miR-615-5p was used to transfect in CAFs. (**K**) Representative image (left panel) and quantitative analysis (right panel) of Miapaca2 cell migration and invasion in the transwell assay with the indicated treatment. Scale bars, 200 μm. (**L**) Quantitative analysis of DRG neurite length per cell body cluster area with the indicated treatment. (**M**) Representative image (left panel) of a co-culture system involving DRG neurons and Miapaca2 cells. Quantitative analysis (right panel) assessing the neuroinvasive potential of Miapaca2 cells with the indicated treatment. Scale bar, 500 μm.*P < 0.05, **P < 0.01, ***P < 0.001, ****P < 0.0001

To determine whether circKLHL24 enhances CXCL12 expression by counteracting miR-615-5p, CAFs were transfected with either a miR-615-5p mimic or inhibitor, followed by qRT-PCR, Western blot and ELISA analysis. CXCL12 mRNA, protein levels and secretion levels were significantly reduced in the miR-615-5p mimic group, but elevated in the inhibitor group, indicating that miR-615-5p negatively regulates CXCL12 expression (Fig. 7A-F). Given that microRNAs typically suppress target gene expression by binding to the 3’ untranslated regions (UTRs), sequence analysis identified a potential miR-615-5p binding site in the CXCL12 3’ UTR (Fig. 7G). Luciferase reporter assays confirmed that miR-615-5p significantly reduced luciferase activity in the wild-type CXCL12 3’ UTR construct, whereas mutation of the binding site abolished this effect, demonstrating that miR-615-5p directly targets the CXCL12 3’ UTR to mediate its degradation (Fig. 7G). Importantly, miR-615-5p upregulation attenuated the ability of circKLHL24 to enhance CXCL12 expression and secretion in CAFs, further supporting the role of miR-615-5p in regulating CXCL12 levels (Fig. 7H and J). Functionally, circKLHL24-overexpressing CAFs lost part of their ability to promote tumor cell invasion, migration, and neurite outgrowth upon miR-615-5p upregulation, indicating that miR-615-5p acts as a functional mediator of circKLHL24’s effect on CXCL12 (Fig. 7K and M).

To further evaluate the feasibility and functional contribution of this dual-pathway model, we performed a series of rescue experiments using circKLHL24 constructs with mutations in either or both binding sites. Mutation of the Sec31A-binding region (circKLHL24-ΔSec) led to a significant reduction in CXCL12 secretion, which could be partially rescued by Sec31A overexpression (Figure S10C). Similarly, disruption of the miR-615-5p binding site (circKLHL24-ΔmiR) also diminished CXCL12 secretion, while co-treatment with a miR-615-5p inhibitor restored it to a comparable extent (Figure S10D). Notably, double-site mutation (circKLHL24-ΔSec&ΔmiR) resulted in an even more pronounced decrease in CXCL12 secretion. Rescue of only one axis (either OE-Sec or miR-615-5p inhibitor) led to partial recovery, while simultaneous restoration of both axes achieved near-complete rescue of CXCL12 secretion (Figure S10E). Together, these findings suggest that circKLHL24 enhances CXCL12 expression by antagonizing miR-615-5p-mediated suppression.

### circKLHL24/CXCL12 axis drives perineural invasion in pancreatic cancer in vivo

**Fig. 8:**
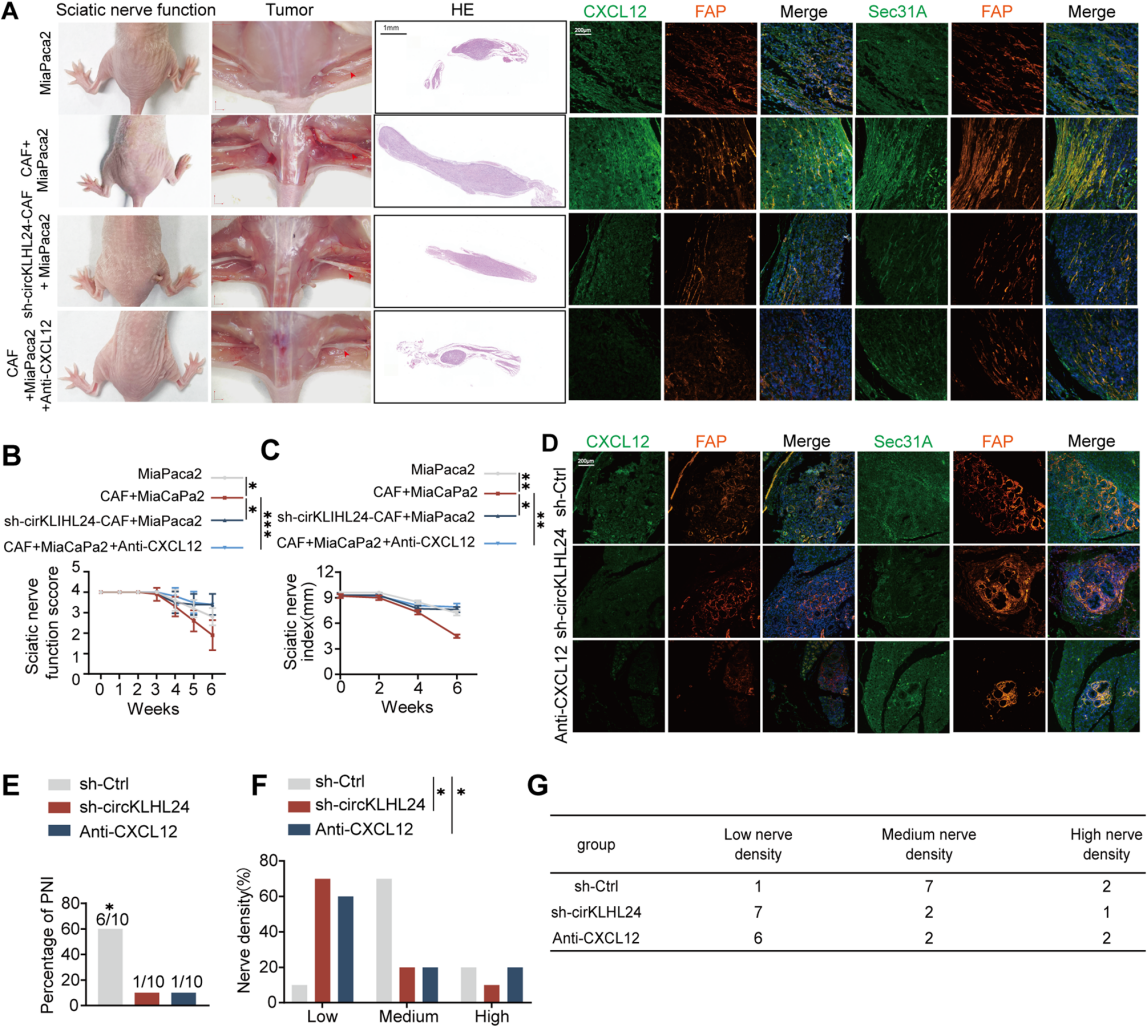
circKLHL24/CXCL12 Axis Drives Perineural Invasion in Pancreatic Cancer In Vivo. (**A**) Gross and surgical images, H&E staining and mIF images. mIF showed CXCL12, FAP and Sec31A expression in the sciatic nerve invasion model. FAP served as a CAF marker. HE Scale bars, 1 mm. mIF Scale bars, 200 μm. (**B-C**) Sciatic nerve function scores and sciatic nerve indexes of mice treated as indicated. (*n* = 10 mice per group; Kruskal-Wallis test with Dunn’s multiple comparisons test; data are shown as the means ± SD). (**C-G**) KPC mice (LSL-KRAS^G12D/+^; LSL-TP53^R172H^/^+^; PDX-1-CRE^+/+^) were divided into three groups. Mice were randomly assigned into three groups. Mice were injected with AAV-packaged shNC (AAV-shNC) (*n* = 10) or with AAV-packaged shcircKLHL24 (AAV-shcircKLHL24) ( *n* = 10) into the tail vein at a dose of 7 × 10^11^ viral genomes (vg) per mouse. The third group received intraperitoneal (i.p.) injection of anti-CXCL12 (*n* = 10),40 mg/kg, once every three days. (**D**) Representative mIF images showing PNI status and CXCL12, Sec31A, FAP, expression in KPC mice treated as indicated. FAP served as a CAF marker. (**E**) Proportion of PNI in KPC mice under the specified treatments.(Fisher’s exact test). (**F-G**) Nerve density percentage (left) and number (right) in KPC mice following the indicated treatments. (Chi-square test). *P < 0.05, **P < 0.01, ***P < 0.001

To further investigate the in vivo role of the circKLHL24/CXCL12 axis in perineural invasion, Panc-1 cells were inoculated into the sciatic nerves of nude mice, either alone or in combination with CAFs. Multiplex immunofluorescence (mIF) analysis further revealed increased CXCL12 expression in xenografts co-injected with CAFs (Fig. 8A). Co-injection with CAFs led to more severe hind limb paralysis, accompanied by a notable reduction in hind paw width (Fig. 8B-C). Conversely, circKLHL24 depletion in CAFs significantly reduced the severity of PNI and downregulated CXCL12 expression (Fig. 8A-C). Similarly, neutralizing CXCL12 significantly alleviated PNI severity, as evidenced by reduced hind limb paralysis and preservation of hind paw width (Fig. 8A-C).

To better simulate the interactions among CAFs, tumor cells, and nerves in vivo, we used the KPC mouse model (LSL-KRASG12D/+; LSL-TP53R172H/+; PDX-1-CRE+/+), which develops spontaneous pancreatic cancer. Mice were randomly assigned into three groups and injected with either AAV-packaged sh-NC (sh-NC) or AAV-packaged sh-circKLHL24 (sh-circKLHL24). CircKLHL24 depletion led to a significant reduction in CXCL12 expression, further reinforcing its role in this pathway (Fig. 8D). Furthermore, Depleting circKLHL24 or neutralizing CXCL12 significantly abolished PNI, compared to the negative control group (Fig. 8E). Additionally, intratumoral nerve density was markedly lower in the sh-circKLHL24 and neutralizing CXCL12 groups than in the shNC group (Fig. 8F-G). Collectively, these findings demonstrate that circKLHL24 promotes PNI in pancreatic cancer by upregulating CXCL12, thereby facilitating tumor–nerve interactions in vivo.

### Clinical relevance of the circKLHL24/CXCL12 axis in PDAC

**Fig. 9:**
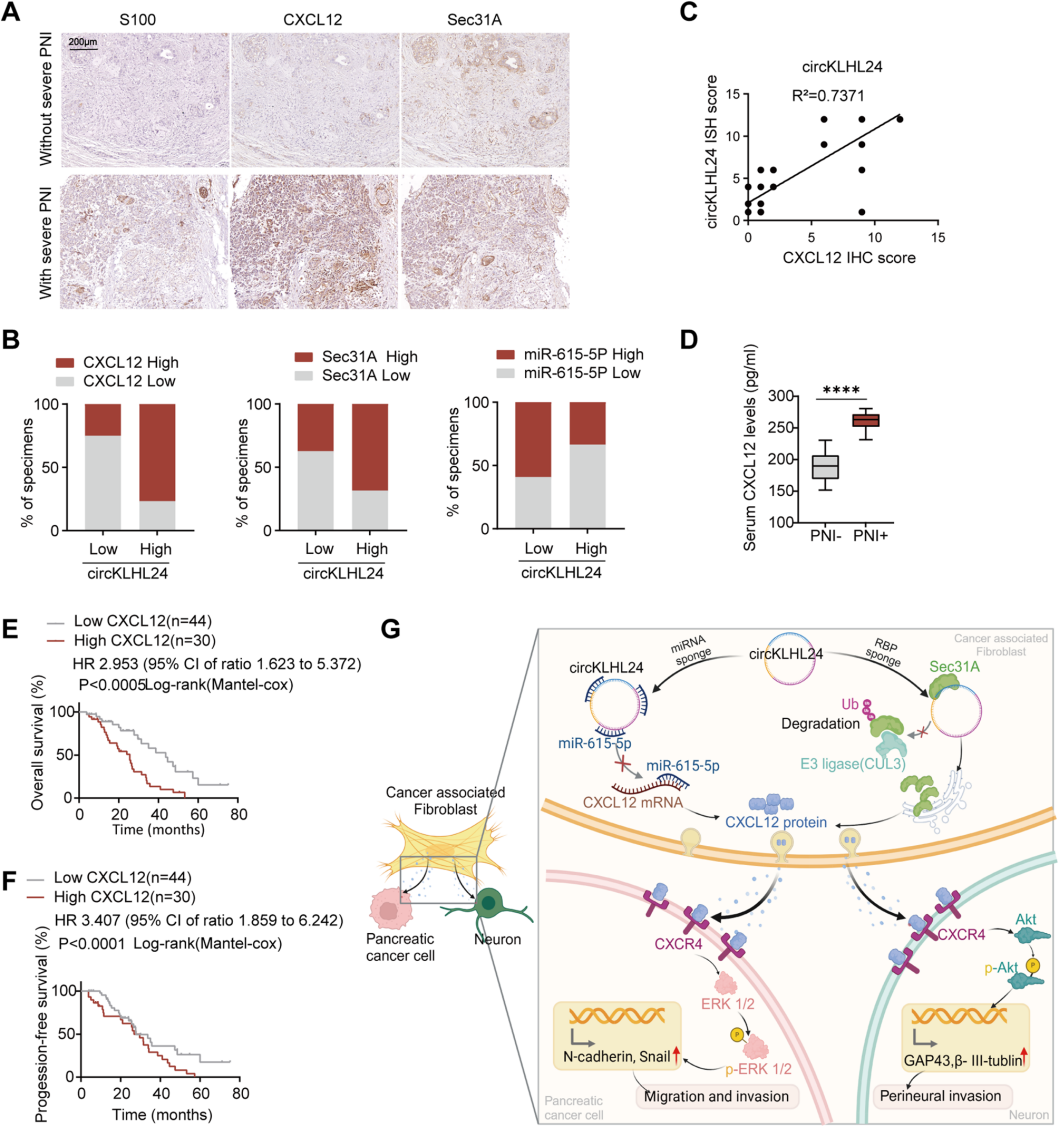
Clinical implication of the circKLHL24/CXCL12 axis in PDAC. (**A**) Representative images of IHC for CXCL12 and Sec31A in PDAC tissues with or without severe PNI. Scale bars, 200 μm. (**B**) Quantification of the percentage of specimens with low or high Sec31A, miR-615-5p and CXCL12 in the low or high circKLHL24 expression groups. (**C**) Correlation analysis of circKLHL24 and CXCL12 expression in PDAC tissues (*n* = 74). (**D**) ELISA analysis of the serum CXCL12 level in PNI- (*n* = 31) and PNI+ (*n* = 43) patients. (**E-F**) Kaplan–Meier survival curves for PDAC patients who had PNI or not with high or low CXCL12 expression. (**G**) Schematic illustration of the mechanism by which circKLHL24 enables CAFs to promote PNI in pancreatic cancer. ****P < 0.0001

To investigate the clinical significance of the circKLHL24/CXCL12 axis in PDAC, IHC analyses were conducted on clinical samples from a cohort of 74 PDAC patients stratified by PNI severity. The findings revealed that patients in the circKLHL24-high group showed elevated CXCL12 and Sec31A expression and reduced miR-615-5p levels, whereas the circKLHL24-low group exhibited the opposite pattern (Fig. 9A-B). Furthermore, a strong positive correlation was observed between circKLHL24 and CXCL12 expression (Fig. 9C). Serum analysis further indicated that patients with severe PNI had significantly higher CXCL12 levels compared to those without severe PNI, reinforcing the link between CXCL12 upregulation and PNI progression (Fig. 9D). Additionally, Kaplan–Meier survival analysis demonstrated that patients with high CXCL12 expression had markedly worse overall survival (OS) and disease-free survival (DFS) compared to those in the CXCL12-low group, highlighting its potential as a prognostic biomarker (Fig. 9E-F). Together, these findings suggest that the circKLHL24/CXCL12 axis plays a key role in PNI progression in PDAC (Fig. 9G).

## Discussion

PNI is a hallmark of PDAC, driving local recurrence, neuropathic pain, and poor survival. Although chemokine-mediated neuro-stromal interactions are implicated in PNI, the role of stromal circRNAs in regulating axon guidance remains poorly understood. Here, we identify CAF-derived circKLHL24 as a key regulator of PNI. Our findings outline a CAF-centric circuit where circKLHL24 drives CXCL12 hypersecretion via two complementary mechanisms: stabilizing Sec31A to enhance CXCL12 vesicular trafficking, and sequestering miR-615-5p to boost CXCL12 transcription. This dual regulation fosters a neurotrophic niche, promoting tumor cell–nerve co-invasion through CXCR4-dependent cancer cell invasion and neuronal regeneration. By connecting circRNA regulation, CAF secretome dynamics, and neural plasticity, we recast PNI as a stroma-coordinated process. Clinically, the circKLHL24–Sec31A/CXCL12 axis offers a promising therapeutic avenue, highlighting the translational potential of targeting stromal pathways in pancreatic cancer.

While circRNAs have emerged as key epigenetic regulators in cancer cells, their capacity to reprogram stromal ecosystems, particularly through secretory network modulation, remains underexplored [20]. Building on our systematic analysis of CAF-derived circRNAs in pancreatic cancer—including circCUL2, which promotes an inflammatory CAF phenotype via MyD88-dependent NF-κB signaling [15]; circFARP1, which drives gemcitabine resistance through the LIF/STAT3 axis [16]; and circBIRC6, which contributes to platinum resistance via SUMOylation modulation [17]. We now identify circKLHL24 as the first stromal circRNA that governs neural invasion dynamics. Our integrated analysis of stromal RNA-sequencing data (GSE172096) revealed circKLHL24 as a CAFs-enriched transcript positively correlating with PNI severity in PDAC specimens. Functional validation demonstrated that CAFs-specific circKLHL24 ablation suppresses tumor cell invasiveness and neuron-driven axonogenesis in vitro, while AAV-mediated circKLHL24 silencing in KPC mice significantly reduced neural infiltration. These findings redefine PNI as a stroma-directed process, with CAFs exploiting circRNA-driven secretory programs to hijack neural developmental pathways.

CXCL12, a pleiotropic cytokine binding to its cognate receptor CXCR4, orchestrates tumor-stroma crosstalk by regulating immune evasion, chemoresistance, and metastatic niche formation across multiple malignancies [21, 22]. In pancreatic cancer, CXCL12 not only drives tumor growth, invasion, and immune evasion via CXCR4 signaling, but its clinical blockade also shows promise in enhancing immunotherapeutic efficacy and improving patient outcomes [23]. Our study shows that circKLHL24 localizes predominantly in the cytoplasm of CAFs, where it regulates both the expression and secretion of the key cytokine CXCL12. Emerging clinical strategies aim to disrupt the CXCL12-CXCR4 axis to overcome stromal-driven therapy resistance. A phase IIa trial (NCT02826486) showed that combining the CXCR4 antagonist BL-8040 (motixafortide) with pembrolizumab and chemotherapy achieved a 32% objective response rate and notable survival gains in metastatic PDAC [24]. Additionally, the OPERA trial in advanced pancreatic cancer demonstrated that inhibiting CXCL12 with NOX-A12 plus PD-1 blockade was safe, prolonged treatment duration, and promoted T cell infiltration. These findings underscore CXCL12 pathway modulation as a key component of next-generation stroma-targeted regimens [12]. Our previous work shows that CAF-derived circFARP1 enhances LIF secretion to drive gemcitabine resistance via the LIF/STAT3 axis [16], while other studies confirm that CAF-derived LIF also significantly promotes perineural invasion in PDAC [8]. However, whether CXCL12 directly mediates PNI remains unexplored. Our findings of significantly elevated CXCL12 in the PNI microenvironment introduce the circKLHL24/CXCL12 axis as a novel therapeutic target and biomarker warranting further clinical evaluation.

To delineate the molecular interplay of circKLHL24, we performed proteomic profiling and identified Sec31A as a direct interactor critical for PNI. Notably, circKLHL24 physically associates with Sec31A, disrupting its binding to USP8 and thereby inhibiting CXCL12 ubiquitination. As a COPII vesicle component, Sec31A plays pivotal roles in ER-to-Golgi protein trafficking, autophagy, nerve repair, and immune regulation [19, 25–27]. This interaction highlights a novel regulatory axis where circKLHL24 modulates secretory pathways by hijacking Sec31A-mediated transport machinery. Beyond protein stabilization, circKLHL24 operates as a competitive endogenous RNA (ceRNA) to sequester miR-615-5p, a post-transcriptional repressor of CXCL12. Recent advances highlight that circRNAs perform distinct functions across different spatial contexts. For instance, circACTN4 coordinates nuclear YBX1/FZD7 transcriptional activation and cytoplasmic miR-424-5p sponging in cholangiocarcinoma [28]. Similarly, circCCAC1 drives cholangiocarcinoma progression by sponging miR-514a-5p to boost YY1/CALML signaling in tumor cells and by disrupting endothelial integrity via EZH2 sequestration and SH3DGL2 upregulation (29). Notably, we found that circKLHL24 exerts a dual cytoplasmic function by concurrently enhancing CXCL12 expression and secretion, revealing a coordinate circRNA regulatory code that drives neural invasion through tumor microenvironment rewiring.

Although CXCL12 secretion plays a pivotal role in the dynamic remodeling of the tumor microenvironment, its upstream regulatory mechanisms remain poorly understood. The conventional secretory pathway depends on COPII vesicle-mediated transport from the endoplasmic reticulum (ER) to the Golgi, with Sec31A serving as a core scaffold protein that directly mediates vesicle budding and cargo sorting. In the TME, CXCL12 secretion is highly heterogeneous—tumor cells engage in autocrine CXCR4 signaling to drive metastasis, whereas stromal cells like CAFs secrete it paracrinally to promote immunosuppression, angiogenesis, and neural remodeling [30–32]. Our work is the first to demonstrate that, in CAFs, Sec31A acts as a critical rate-limiting factor for CXCL12 secretion by directing its transport from the ER to the Golgi, thereby ensuring efficient extracellular release. Moreover, our mechanistic studies reveal that loss of Sec31A results in CXCL12 retention within the ER and a marked decrease in its Golgi localization, suggesting that Sec31A may selectively recognize specific sorting signals—such as transmembrane domains or glycosylation modifications—in CXCL12 [19, 33]. This observation aligns with the classical model in which COPII components, including the Sect. 24 family, orchestrate cargo sorting [34]. While prior research on Sec31A has predominantly focused on its metastasis-promoting functions—such as driving lung metastasis by modulating collagen secretion [35] and enhancing mesenchymal stem cell osteogenesis by sustaining autophagy [19]. Our study unveils a novel link between Sec31A and the neural invasion microenvironment, demonstrating that Sec31A facilitates perineural invasion by regulating CXCL12 secretion. Notably, our data show that circKLHL24 directly interacts with Sec31A, competitively interfering with its interaction with CUL3 and thereby preventing Sec31A degradation. This circRNA–protein interaction extends the functional repertoire of circRNAs beyond the traditional ceRNA paradigm, unveiling a novel epigenetic mechanism that regulates the secretory machinery.

From a clinical perspective, targeting the CXCL12 secretion pathway presents a dual advantage. Inhibiting upstream regulators such as Sec31A or circKLHL24 may circumvent the compensatory signaling often observed with direct blockade of the CXCL12–CXCR4 axis, while the stromal-specific regulation of Sec31A offers an opportunity for selective intervention in the tumor neural microenvironment. Accordingly, the development of small-molecule inhibitors targeting the circKLHL24/Sec31A interface or AAV-mediated Sec31A degraders could potentially enhance the efficacy of existing CXCR4 antagonists or immune checkpoint inhibitors. Furthermore, our preliminary multicenter cohort data indicate that Sec31A protein levels in CAFs are significantly inversely correlated with PNI and patient survival, suggesting that the Sec31A–CXCL12 axis might serve as a predictive biomarker for PNI risk.

## Conclusions

In summary, our study uncovers a novel CAF-derived molecular axis that orchestrates cancer–stroma crosstalk to drive PNI. We demonstrate that circKLHL24 simultaneously stabilizes Sec31A to promote CXCL12 vesicular trafficking and sponges miR-615-5p to enhance its transcription, thereby establishing a neurotrophic niche that promotes CXCR4-driven tumor invasion and nerve outgrowth. This dual regulatory mechanism not only redefines PNI as a stroma-coordinated process but also highlights the clinical innovation of targeting the CAF–tumor cell dialogue. Collectively, our work advocates a “stromal decoupling” strategy that pairs circRNA-based interventions with neuro-microenvironment modulation, surpassing the traditional tumor cell–centric paradigm.

## Supplementary Information

Below is the link to the electronic supplementary material.

Supplementary Material 1

Supplementary Material 2

Supplementary Material 3

Supplementary Material 4

Supplementary Material 5

Supplementary Material 6

Supplementary Material 7

Supplementary Material 8

Supplementary Material 9

Supplementary Material 10

Supplementary Material 11

Supplementary Material 12

Supplementary Material 13

Supplementary Material 14

Supplementary Material 15

Supplementary Material 16

Supplementary Material 17

## Data Availability

Sequencing data has been uploaded to GEO (GSE172096, GSE299209), other data can be obtained from the manuscript and its supplementary files, or provided upon request from the corresponding author.

## Abbreviations

CAFs: Cancer-associated fibroblasts
circRNAs: Circular RNAs
qRT-PCR: Real-time PCR
PNI: Perineural invasion
CXCL12: Chemokine (C-X-C Motif) ligand 12
PDAC: Pancreatic ductal adenocarcinoma
TME: Microenvironment
LIF: Leukemia inhibitory factor
DFS: Disease-free survival
OS: Overall survival
DRG: Dorsal root ganglion
KEGG: Kyoto encyclopedia of genes and genomes
FISH: Fluorescence in situ hybridization
RIP: RNA immunoprecipitation
CHX: Cycloheximide
ER: Endoplasmic reticulum
ceRNA: Competitive endogenous RNA
