# Supplementary figures and images for: Cancer-associated fibroblast-derived circKLHL24 drives perineural invasion in pancreatic cancer via dual regulation of the sec31a-CXCL12 axis
Source: J Exp Clin Cancer Res. 2025 Oct 7;44:281. doi: 10.1186/s13046-025-03489-2 (PMC12502155; doi:10.1186/s13046-025-03489-2)

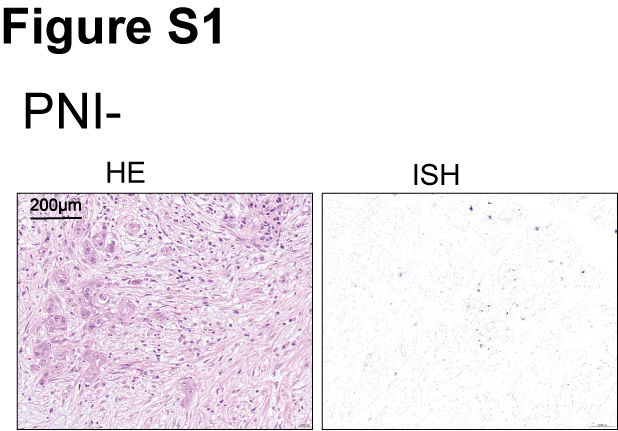

Supplement: Supplementary file 1 — Supplementary Material 1 [file 13046_2025_3489_MOESM1_ESM.tif]

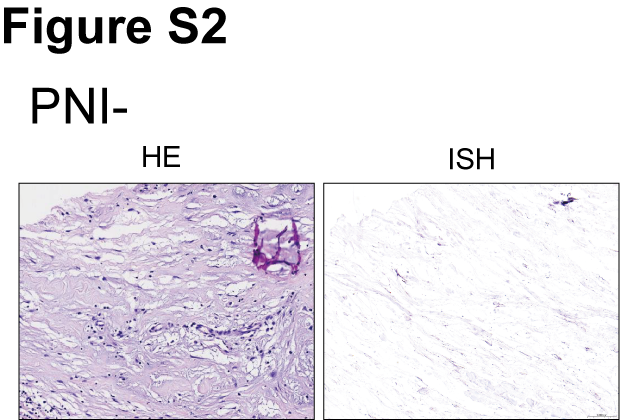

Supplement: Supplementary file 2 — Supplementary Material 2 [file 13046_2025_3489_MOESM2_ESM.tif]

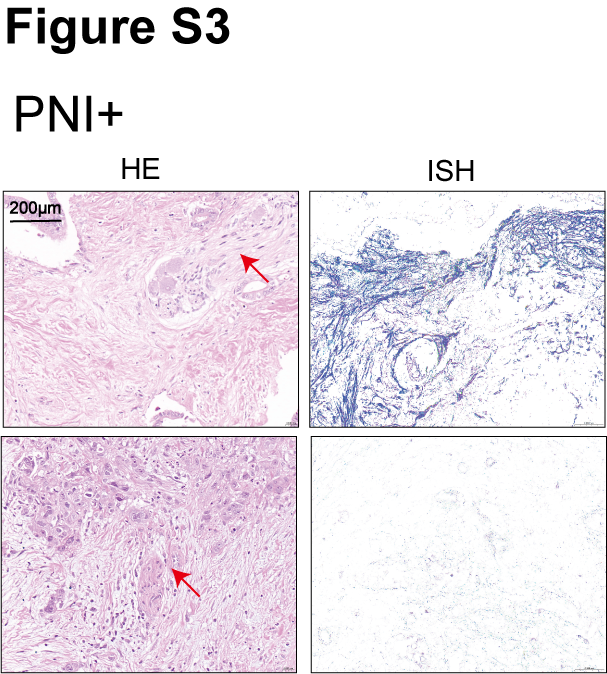

Supplement: Supplementary file 3 — Supplementary Material 3 [file 13046_2025_3489_MOESM3_ESM.tif]

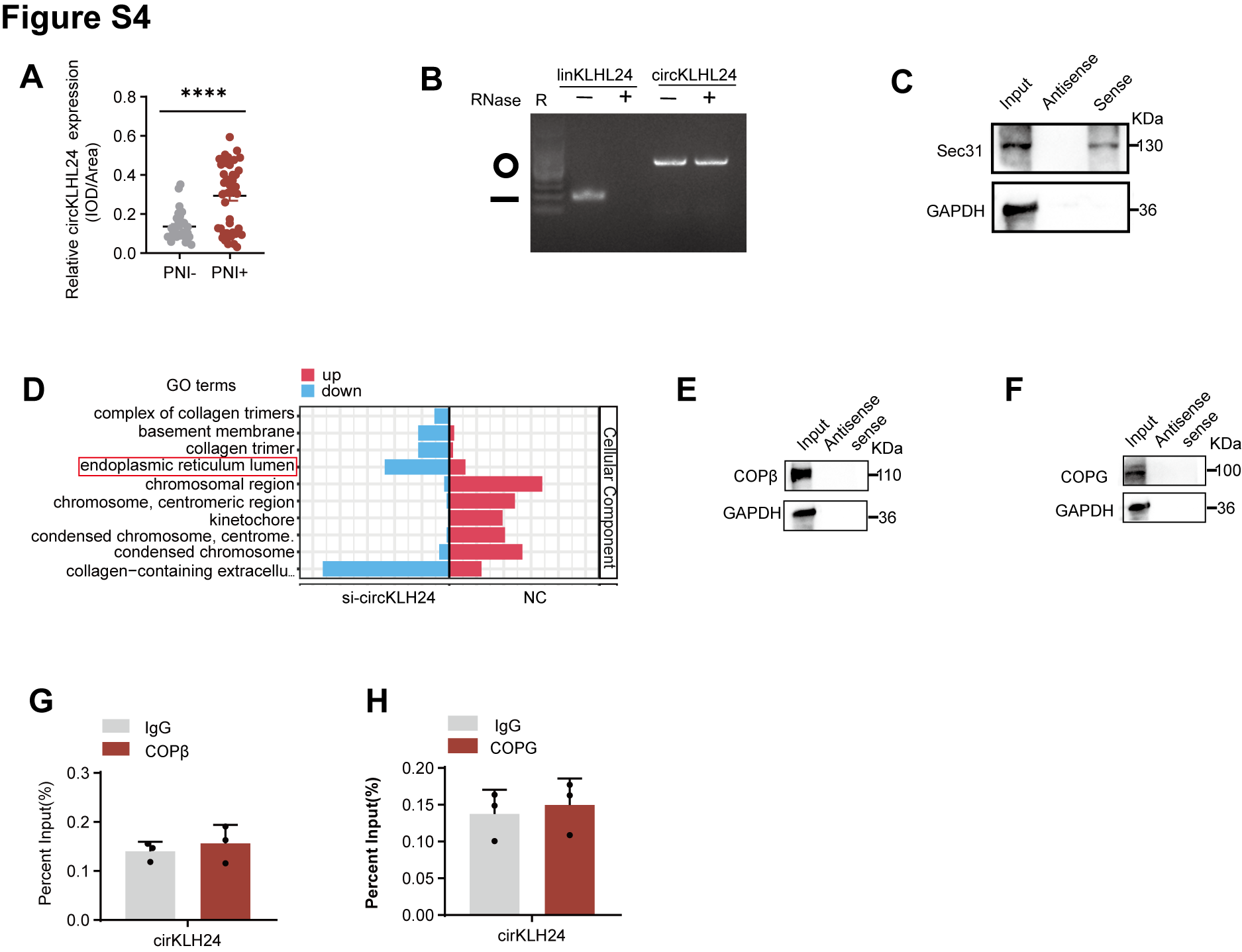

Supplement: Supplementary file 4 — Supplementary Material 4 [file 13046_2025_3489_MOESM4_ESM.tif]

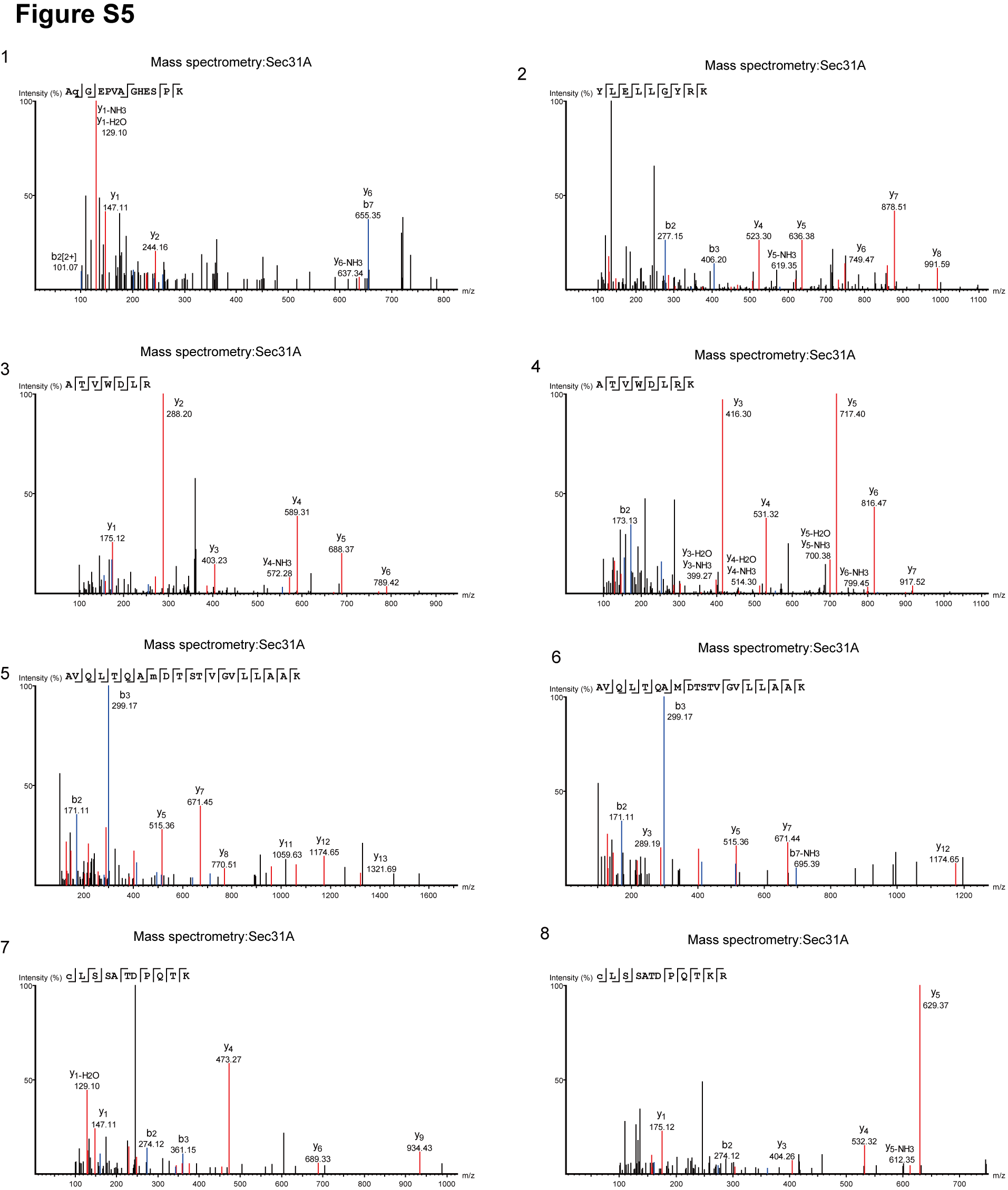

Supplement: Supplementary file 5 — Supplementary Material 5 [file 13046_2025_3489_MOESM5_ESM.tif]

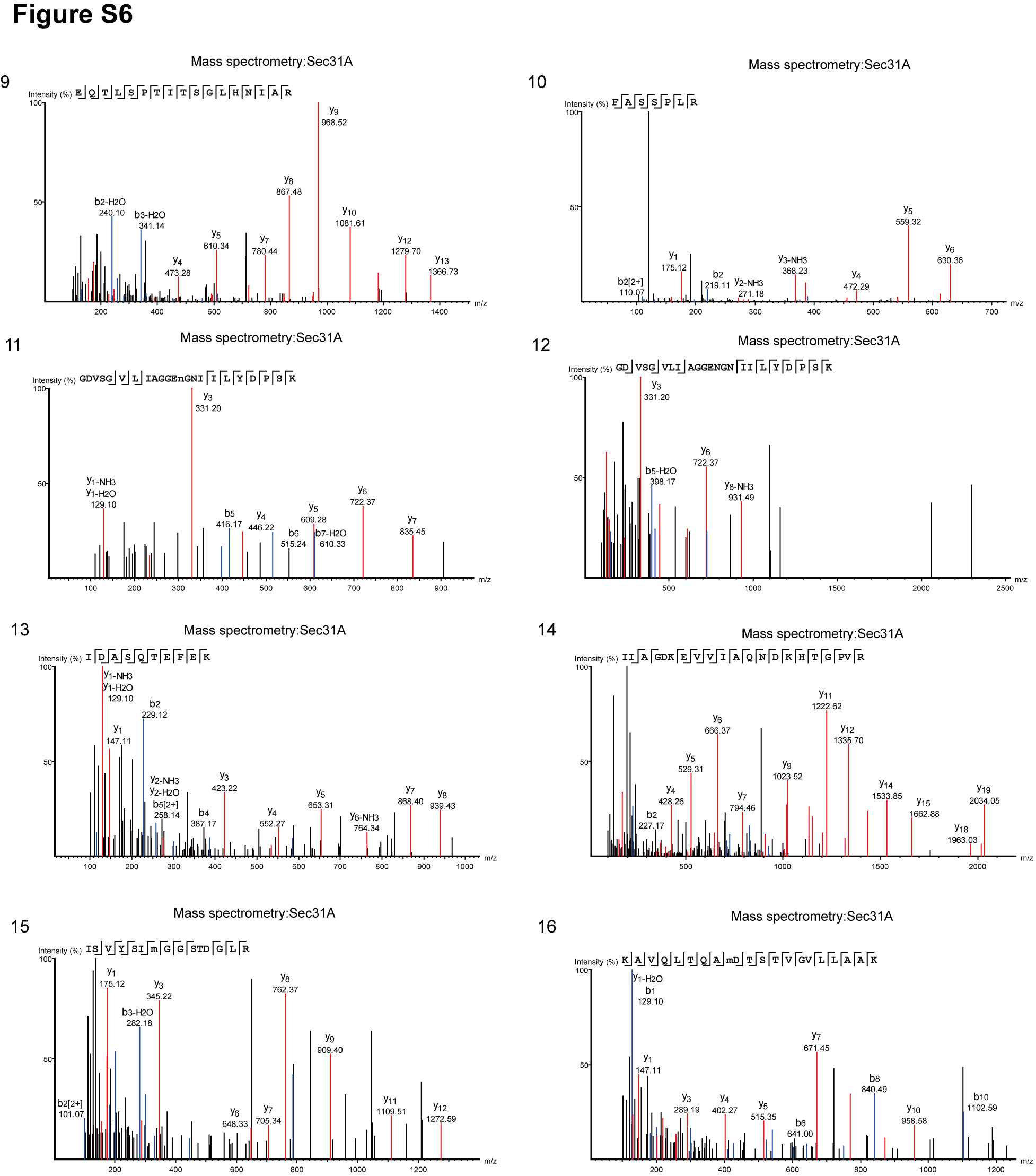

Supplement: Supplementary file 6 — Supplementary Material 6 [file 13046_2025_3489_MOESM6_ESM.tif]

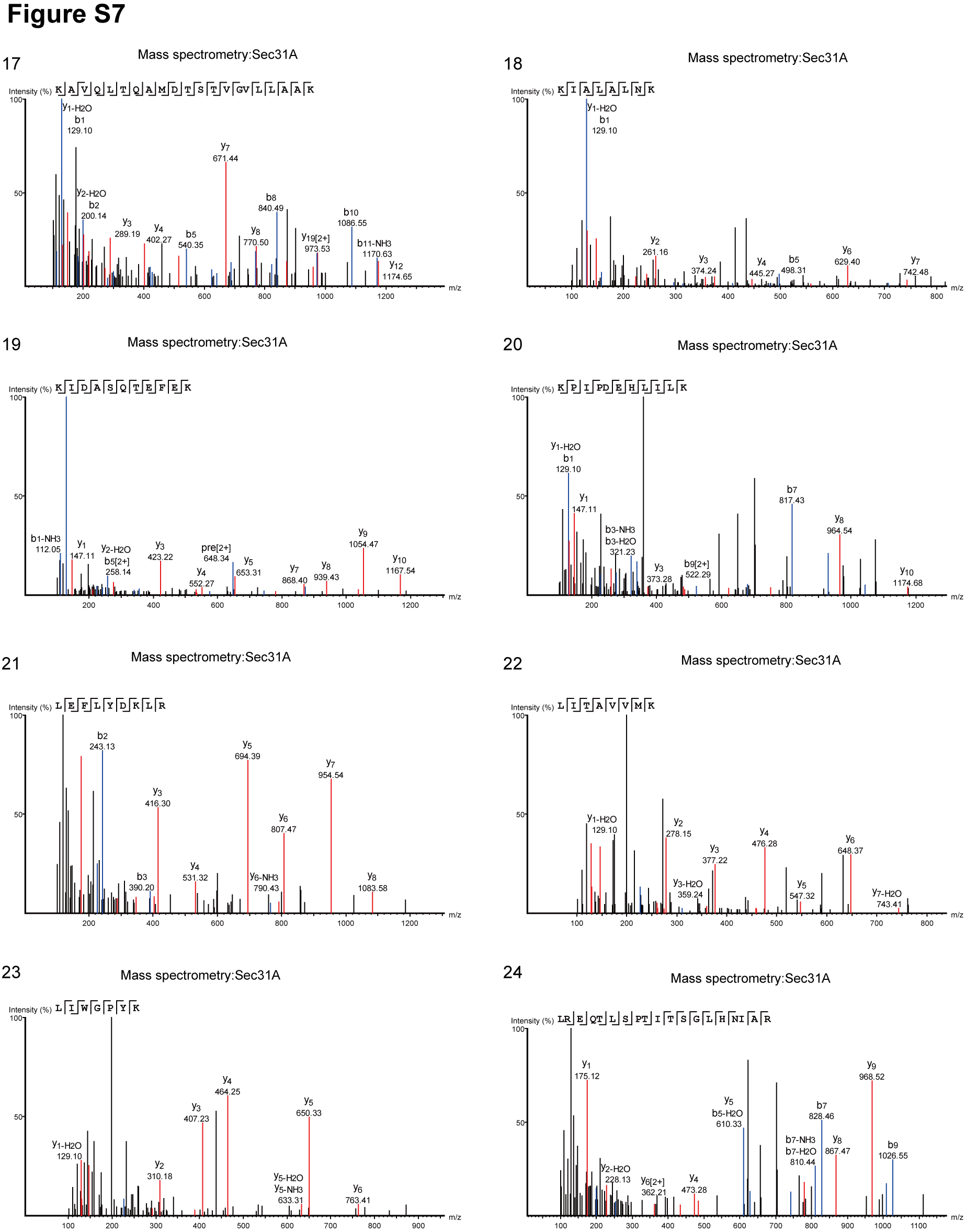

Supplement: Supplementary file 7 — Supplementary Material 7 [file 13046_2025_3489_MOESM7_ESM.tif]

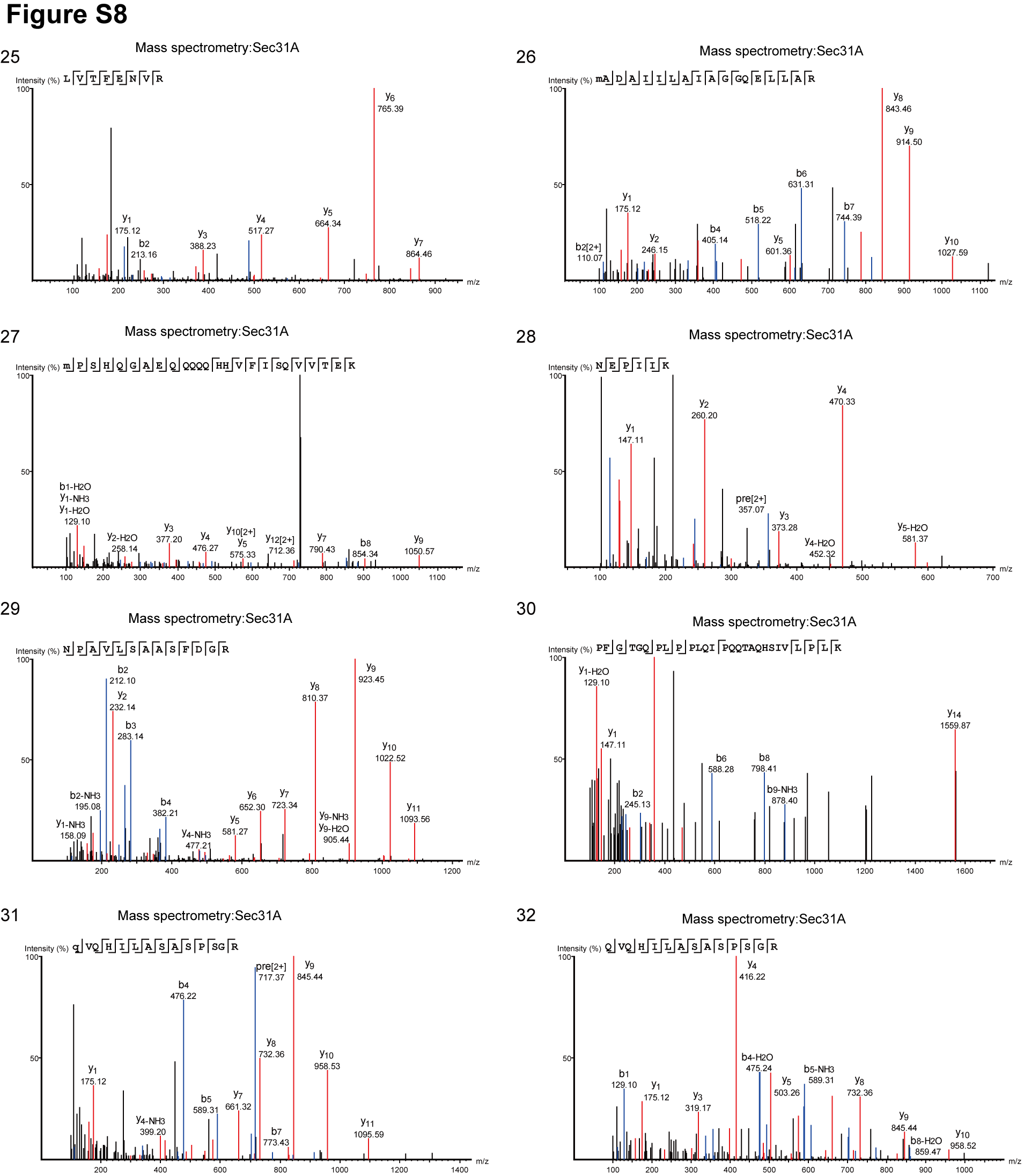

Supplement: Supplementary file 8 — Supplementary Material 8 [file 13046_2025_3489_MOESM8_ESM.tif]

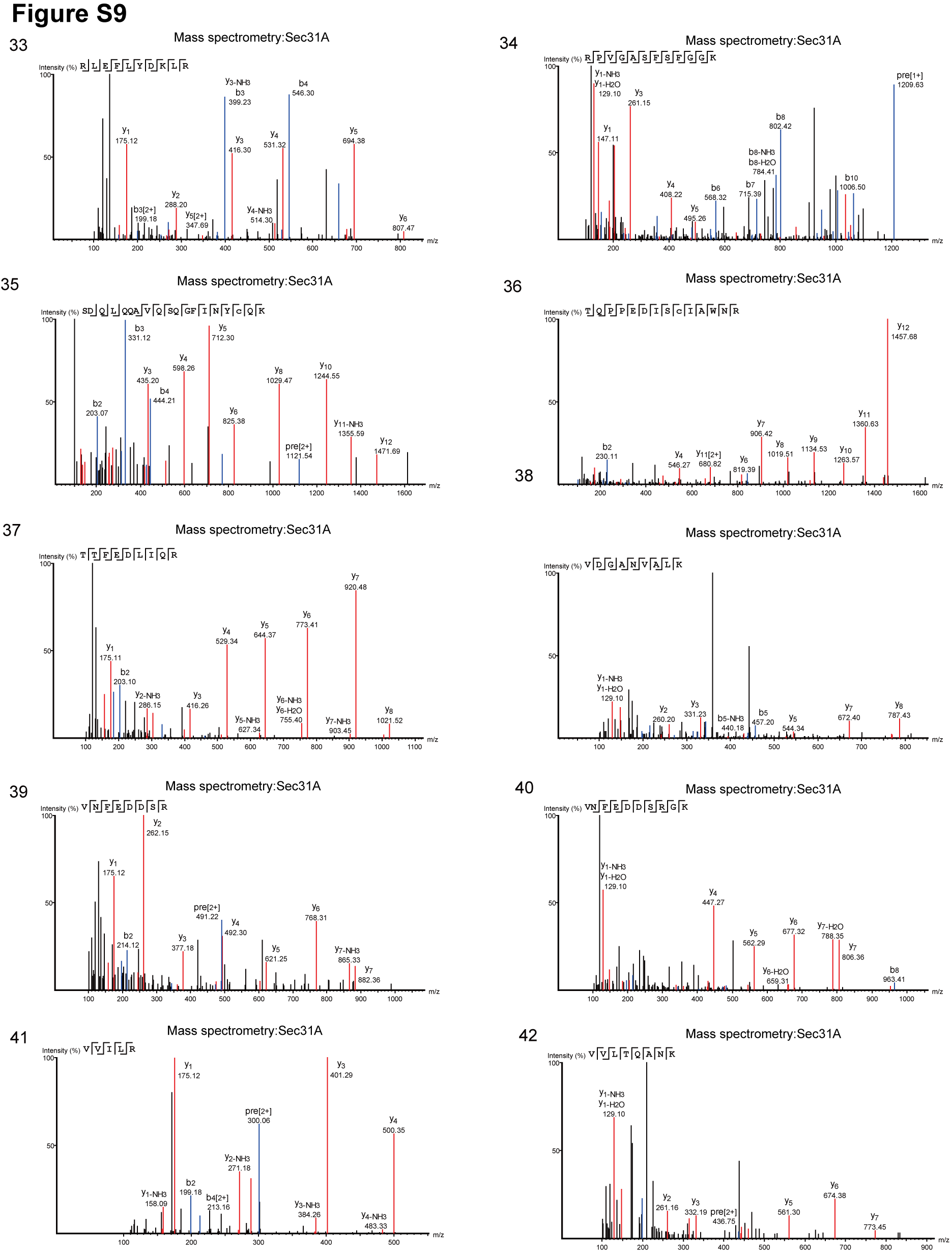

Supplement: Supplementary file 9 — Supplementary Material 9 [file 13046_2025_3489_MOESM9_ESM.tif]

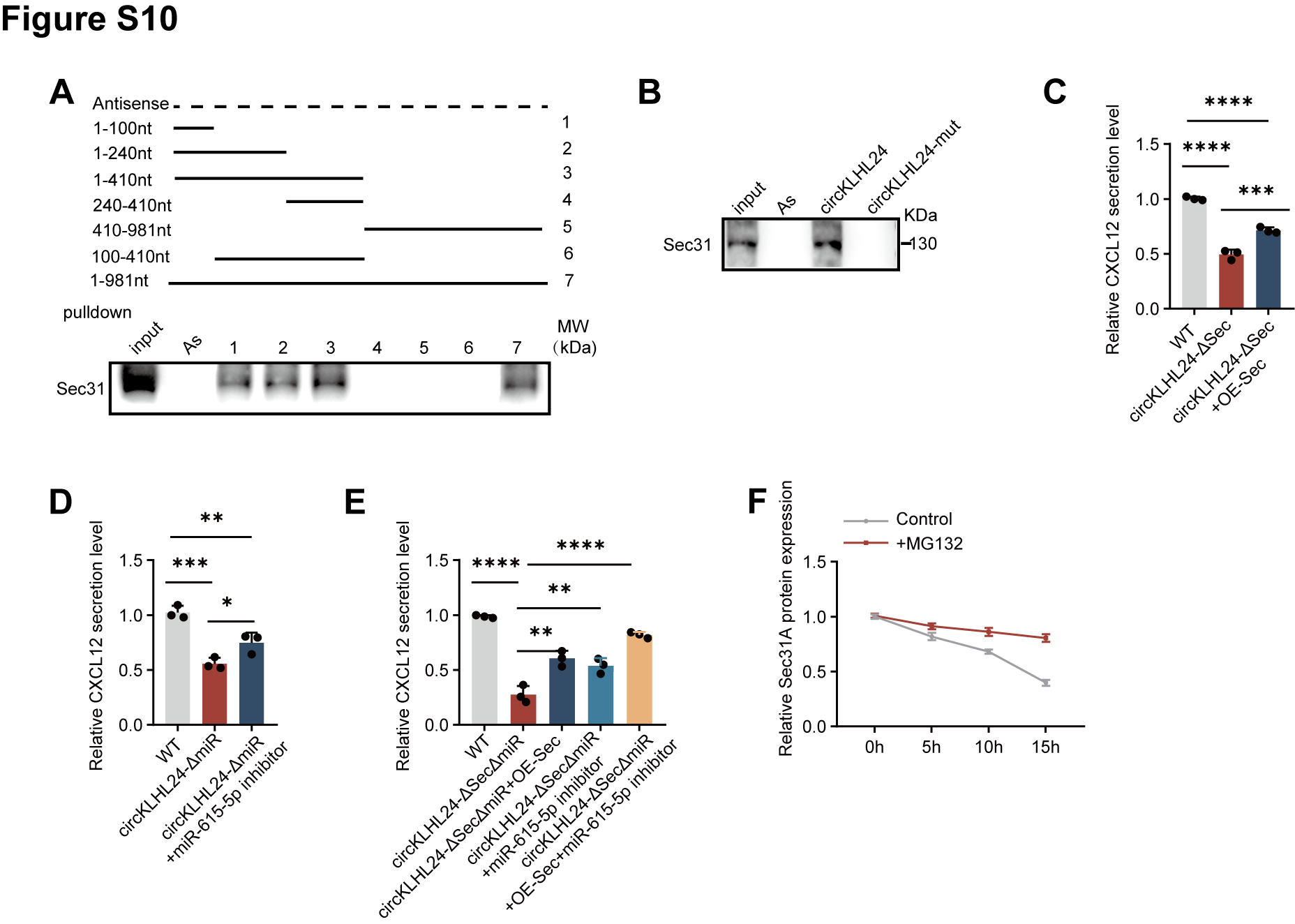

Supplement: Supplementary file 10 — Supplementary Material 10 [file 13046_2025_3489_MOESM10_ESM.tif]

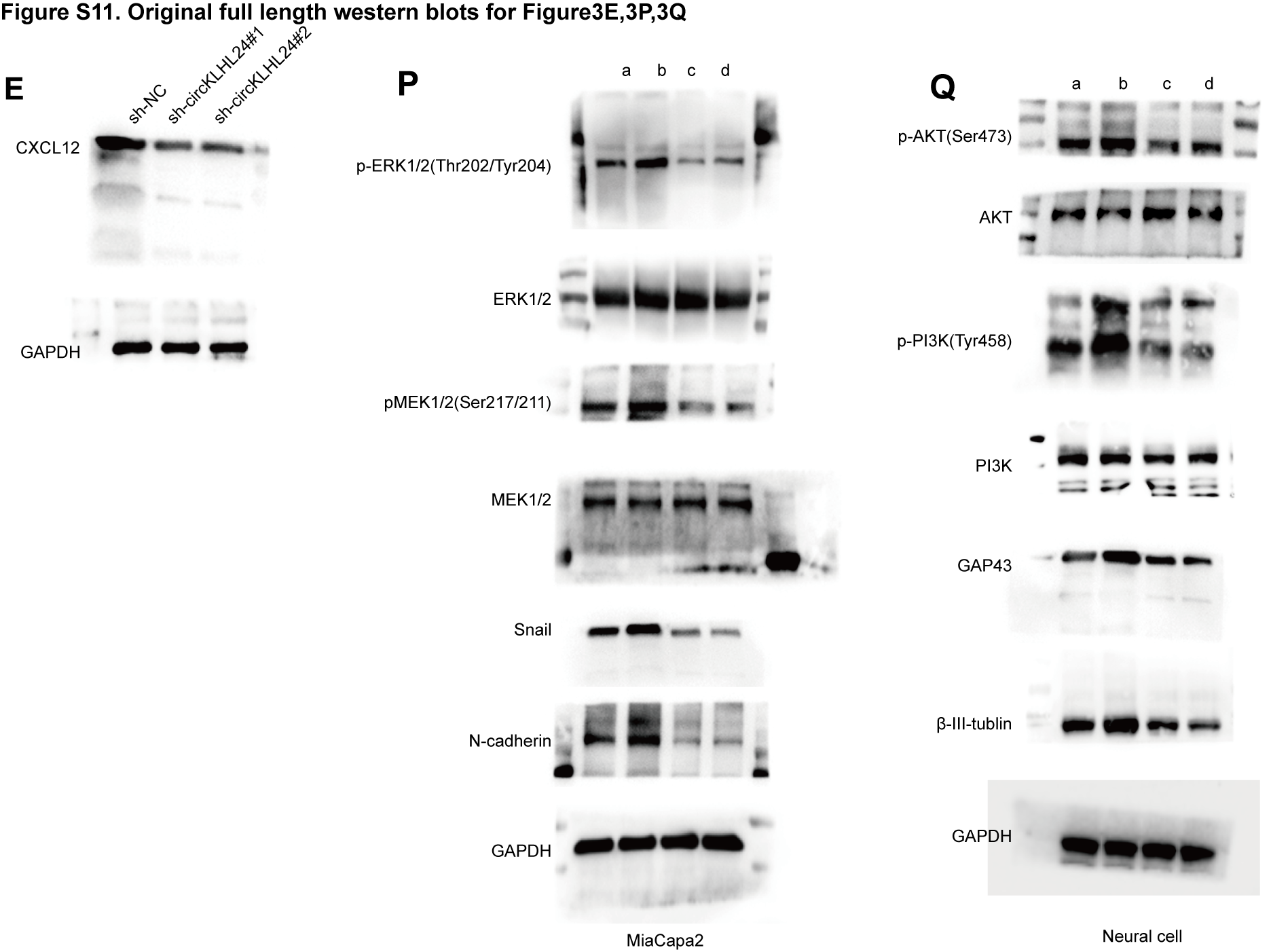

Supplement: Supplementary file 11 — Supplementary Material 11 [file 13046_2025_3489_MOESM11_ESM.tif]

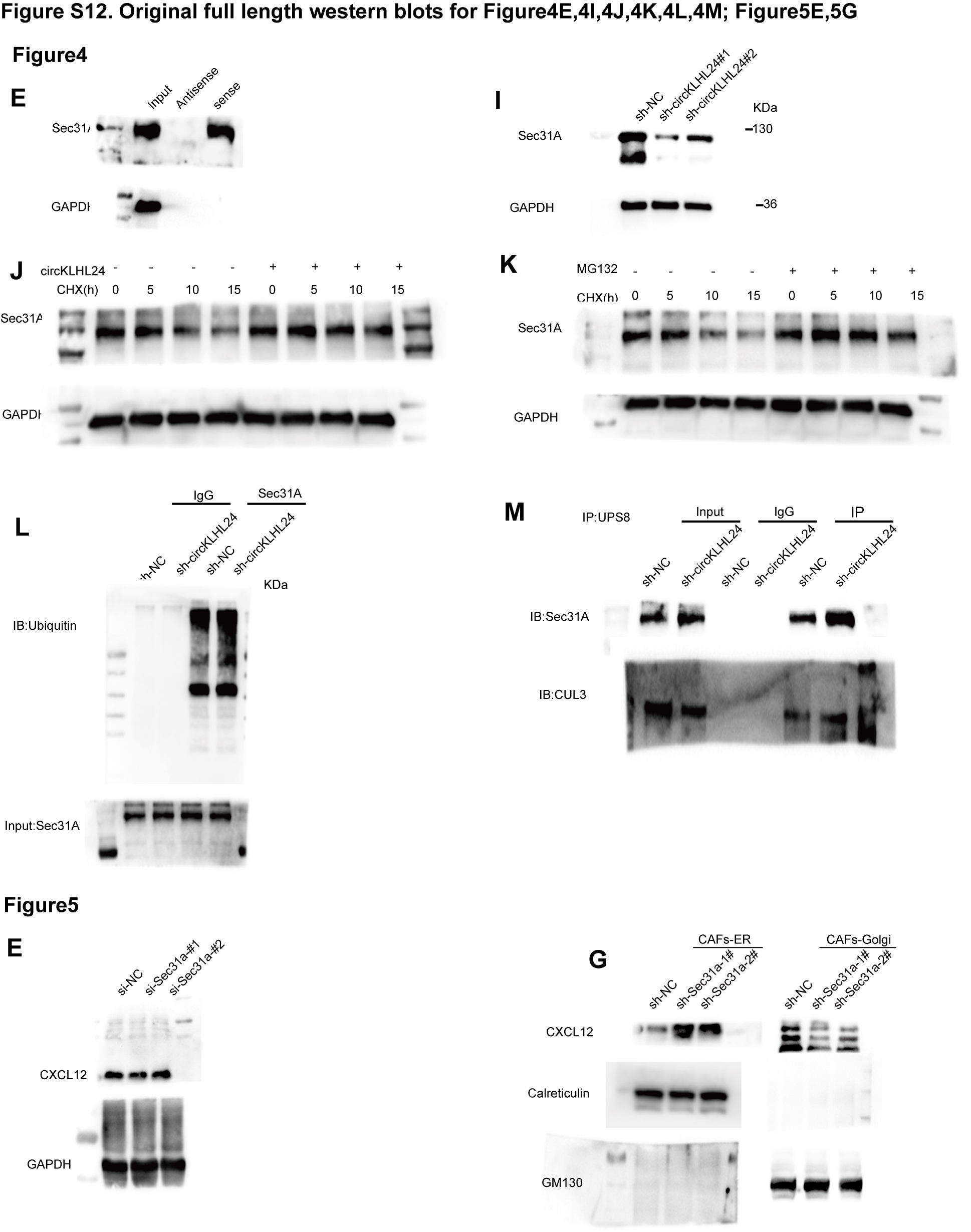

Supplement: Supplementary file 12 — Supplementary Material 12 [file 13046_2025_3489_MOESM12_ESM.tif]

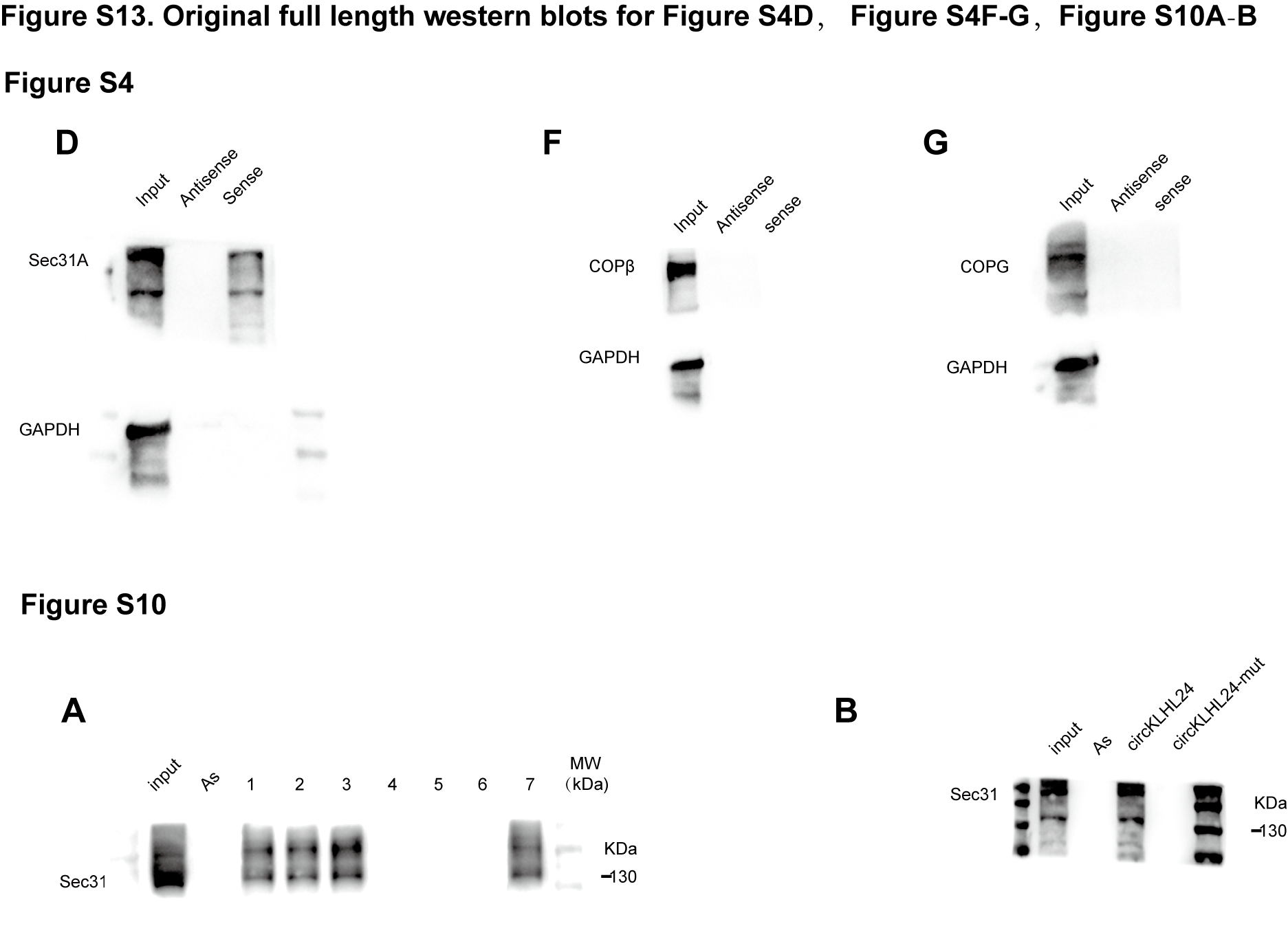

Supplement: Supplementary file 13 — Supplementary Material 13 [file 13046_2025_3489_MOESM13_ESM.tif]
