# Supplementary material for: Cancer-associated fibroblast-derived circKLHL24 drives perineural invasion in pancreatic cancer via dual regulation of the sec31a-CXCL12 axis
Source: J Exp Clin Cancer Res. 2025 Oct 7;44:281. doi: 10.1186/s13046-025-03489-2 (PMC12502155; doi:10.1186/s13046-025-03489-2)
Supplement: Supplementary file 14 — Supplementary Material 14 [file 13046_2025_3489_MOESM14_ESM.docx]

**Supplemental Table S1. Antibody used in this study.**

| Antibody | Cat no. | RRID number |
| --- | --- | --- |
| Anti-GAPDH antibody | ab181602, abcam | AB_181602 |
| Anti-S100 antibody | ab4066, abcam | AB_4066 |
| MEK1/2 Polyclonal antibody | 11049-1-AP, proteintech | AB_2140649 |
| ERK1/2 | 11257-1-AP，proteintech | AB_2139822 |
| Phospho-MEK1/2 (Ser217/221) | # 9154，CST | AB_2138017 |
| Phospho-p44/42 MAPK (Erk1/2) (Thr202/Tyr204) | #4370，CST | AB_2315112 |
| SNAI1 Polyclonalantibody | 13099-1-AP，proteintech | AB_2191756 |
| N-cadherin Polyclonal antibody | 22018-1-AP，proteintech | AB_2813891 |
| GAP43 Polyclonal antibody | 16971-1-AP，proteintech | AB_2278881 |
| β3-Tubulin (D71G9) Rabbit mAb | #5568，CST | AB_10694505 |
| PI3 Kinase p85 Alpha Monoclonal antibody | 60225-1-Ig , proteintech | AB_11042594 |
| Phospho-PI3 Kinase p85 (Tyr458)/p55 (Tyr199) (E3U1H) Rabbit mAb | 17366, CST | AB_2895293 |
| FAP (F1A4G) Rabbit mAb | 52818S , CST | AB_3674735 |
| CXCL12/SDF-1 Polyclonal antibody | 17402-1-AP, proteintech | AB_2878404 |
| SEC31A Polyclonal antibody | 17913-1-AP,, proteintech | AB_2186378 |
| AKT Polyclonal antibody | 10176-2-AP, proteintech | AB_2224574 |
| Phospho-AKT (Ser473) Monoclonal antibody | 66444-1-Ig, proteintech | AB_2782958 |
