# Supplementary material for: Cancer-associated fibroblast-derived circKLHL24 drives perineural invasion in pancreatic cancer via dual regulation of the sec31a-CXCL12 axis
Source: J Exp Clin Cancer Res. 2025 Oct 7;44:281. doi: 10.1186/s13046-025-03489-2 (PMC12502155; doi:10.1186/s13046-025-03489-2)
Supplement: Supplementary file 15 — Supplementary Material 15 [file 13046_2025_3489_MOESM15_ESM.docx]

**Supplemental Table S2. Primers used in PCR.**

|  | Sequence (5’-3’) |
| --- | --- |
| KLHL24 | F-TGATTGGTGGAGGACCTGATG |
|  | R-CCAAGAATTGGTTTCTGGATCAT |
| CXCL12 | F-TCAGCCTGAGCTACAGATGC |
|  | R-CTTTAGCTTCGGGTCAATGC |
| U6 | F-CTCGCTTCGGCAGCACA |
|  | R-AACGCTTCACGAATTTGCGT |
| cirKLHL24(convergent) | F- TTTGAAGCCGTCATGCGTTG |
| cirKLHL24 (convergent) | R-GTTGGGATGCAACAGAGGGA |
| cirKLHL24(divergent) | F-TGTTGCATGAAGCAAGACGG |
| cirKLHL24(divergent) | R-CTTCTTTATGTGGCTGCGTGG |
| GAPDH(convergent) | F-GTCATCCCTGAGCTGAACGG |
| GAPDH-R (convergent) | R-GTCAAAGGTGGAGGAGTGGG |
| GAPDH(divergent) | F-CACCACACTGAATCTCCCCT |
| GAPDH(divergent) | R-ATTTCCTTCCCGGTTGCAAC |
