# Supplementary material for: Cancer-associated fibroblast-derived circKLHL24 drives perineural invasion in pancreatic cancer via dual regulation of the sec31a-CXCL12 axis
Source: J Exp Clin Cancer Res. 2025 Oct 7;44:281. doi: 10.1186/s13046-025-03489-2 (PMC12502155; doi:10.1186/s13046-025-03489-2)
Supplement: Supplementary file 16 — Supplementary Material 16 [file 13046_2025_3489_MOESM16_ESM.docx]

**Supplemental Table S3. siRNA and shNRA Sequence.**

|  | Sequence( 5’-3’) | Application |
| --- | --- | --- |
| sh-cirKLHL24#1 | GGCCACGCAGCCACAUAAAGATT | shRNA |
| sh-cirKLHL24#2 | ACUAGGCCACGCAGCCACAUATT | shRNA |
| Sh-Sec31A | GCTGTCTTATCAGCTGCTTCGTTCAAGAGACGAAGCAGCTGATAAGACAGCTT | shRNA |
| Cy3-circKLHL24 | gATCT(dT-Biotin)TCTTTATg(dT-Biotin)TggCTgCg(dT-Biotin)TggCC(dT-Biotin)TAgTC | FISH |
| double-DIG-circKLHL24 | (Gigoxin)gATCTTCTTTATgTggCTgCgTggCCTAgTC(Gigoxin) | ISH |
